# Evolution and co-evolution of the suck behaviour, a postcopulatory female resistance trait that manipulates received ejaculate

**DOI:** 10.1186/s12915-025-02171-5

**Published:** 2025-03-26

**Authors:** Pragya Singh, Jeremias N. Brand, Lukas Schärer

**Affiliations:** 1 Department of Environmental Sciences, University of Basel, Zoological Institute, Basel, Switzerland; 2 Department of Chemical Ecology, Bielefeld University, Universitätsstr. 25, Bielefeld, 33615 Germany; 3 Department of Tissue Dynamics and Regeneration, Max Planck Institute for Multidisciplinary Sciences, Göttingen, Germany

**Keywords:** Sexual conflict, Sexual selection, Simultaneous hermaphrodite, Co-evolution, Reproductive traits, Postcopulatory mechanisms, Mating behaviour

## Abstract

**Background:**

Sexual conflicts over the post-mating fate of received ejaculate can favour traits in one sex that are costly to the other. Reciprocally mating hermaphrodites face unique challenges as they mate simultaneously in both the male and female role, potentially leading to receipt of unwanted ejaculate. Reciprocal mating can then give rise to postcopulatory female resistance traits that allow manipulation of received ejaculate. A putative example is the suck behaviour, observed in the flatworm genus *Macrostomum*. It involves the sperm recipient placing its pharynx over its own female genital opening and appearing to suck, likely removing received ejaculate after mating. The genus also contains hypodermically inseminating species that presumably exhibit unilateral mating and have not been observed to suck.

**Results:**

Here, we examine the evolution of the suck behaviour in the *Macrostomum* genus, aiming to document the mating behaviour in 64 species. First, we provide videographic evidence that ejaculate is indeed removed during the suck behaviour in a reciprocally mating species, *Macrostomum hamatum*. Next, we show positive evolutionary correlations between the presence, duration and frequency of reciprocal mating behaviour and the suck behaviour, providing clear evidence that the suck behaviour co-evolves with reciprocal mating behaviour. Finally, we show an association between reproductive behaviour and reproductive morphology, suggesting that the reproductive morphology can be used to infer a species’ mating behaviour.

**Conclusions:**

Together, our study demonstrates sexually antagonistic coevolution leading to the evolution of a postcopulatory behavioural trait that functions as a female counter-adaptation allowing individuals to gain control over received ejaculate in a hermaphroditic sexual system.

**Supplementary Information:**

The online version contains supplementary material available at 10.1186/s12915-025-02171-5.

## Background

Sexual conflict is defined as the conflict between the two sexes over their evolutionary interests involving reproduction [1–3]. The primordial cause of sexual conflict is anisogamy, in which the male sex produces more but smaller gametes (called sperm in animals), whereas the female sex produces fewer but larger gametes (called eggs in animals) [4]. Because of this asymmetry, eggs are often a limiting resource for reproductive success, resulting in divergent interests between the sexes [5, 6]. Furthermore, these conflicting interests can give rise to traits expressed by one sex that are costly to the other sex, resulting in antagonistic co-evolution between the sexes [3, 7]. Although work on sexual conflict has primarily focussed on separate-sexed animals, sexual conflict is also pervasive in the lesser-studied hermaphroditic animals [1, 8–11].

Of particular interest is the reproductive biology of simultaneous hermaphrodites (referred to as hermaphrodites hereafter), which involves unique sexual conflicts. For example, there can be conflicts between the mating partners over the sex role exhibited in a mating, namely mating as a sperm donor, a sperm recipient, or both. Depending on the costs and benefits of mating in each role, this may lead to sex role preferences [9, 11]. These are linked to Bateman’s principle, a term coined by Charnov [1], which reflects the notion that there is a “greater dependence of males for their fertility on frequency of insemination” [5]. In his seminal paper, Charnov [1] explored the proposal that Bateman’s principle also applies to hermaphrodites. He concluded that Bateman’s principle would lead to a mating conflict because individuals may often mate primarily to donate rather than to receive sperm. This conflict over the sex roles can be resolved via different mating strategies. One such strategy is reciprocal mating (also called reciprocal copulation), in which the partners simultaneously mate in both the male and female roles. Each sperm donor is thus also a sperm recipient, and although multiple mating offers more opportunities to donate sperm, it may also lead to receipt of unwanted ejaculate from the partners. While this strategy seems like a cooperative conflict resolution, it could shift the conflict from the precopulatory to the postcopulatory arena [11]. In the presence of sperm competition, a donor—in order to secure a greater share of paternity—may often donate more sperm than the recipient requires for fertilisation, thereby potentially causing direct costs, such as a risk of polyspermy [12]. But even if there are no direct costs posed by the received ejaculate, mating with multiple partners—which is probably the norm for most species [13–15]—could lead to the evolution of cryptic female choice [1, 16, 17]. Thus, receipt of excessive or unwanted ejaculate can favour the evolution of female resistance traits that allow postcopulatory control and rejection of the received ejaculate, e.g. via sperm digestion [1].

Female resistance traits can in turn favour the evolution of male persistence traits, including other mating strategies. Such counter-adaptations may allow the sperm donor to either counteract or bypass the female resistance traits, and thereby retain or regain access to the recipient’s eggs [1, 11]. An example of such an alternative mating strategy involves forced unilateral hypodermic insemination (also called hemocoelic insemination [1];). Here one of the partners mates in the male role and donates sperm, while the other mates in the female role, potentially against its interests, and receives sperm hypodermically via a traumatic male copulatory organ [18–20]. With both types of mating strategy, these sexual conflicts could then lead to the evolution of multiple male persistence and female resistance traits (spanning behaviour, morphology and physiology) that act jointly to either gain access to eggs, or to control and reject the received ejaculate, respectively [3]. Therefore, we might expect behavioural mating strategies to be involved in sexually antagonistic coevolution, and thus to be correlated with morphological and/or physiological traits.

A putative example of a behavioural female resistance trait is the suck behaviour, originally documented in the free-living flatworm *Macrostomum lignano* [21]. Studies in this reciprocally mating simultaneous hermaphrodite have shown that matings are often followed by the suck behaviour, during which the worm bends down and places its pharynx over its own female genital opening (which is connected to the female antrum, the sperm-receiving organ) and then appears to suck. The suck behaviour is hypothesised to be a postcopulatory behaviour used to remove sperm or other ejaculate components received during mating, thereby functioning as a female resistance trait [22]. However, despite multiple studies on this behaviour [21, 23–27], there is currently no direct evidence that it actually removes sperm and/or ejaculate. Moreover, if the suck behaviour functions as a postcopulatory sexual selection process, it could affect the strength of sperm competition and potentially impact the optimal sex allocation (i.e. the amount of resources allocated to the male and female function) [28, 29]. Indeed, studies have documented both inter- and intra-specific variation in sex allocation in *Macrostomum* [30–32], with mating behaviour predicting the evolution of a species’ sex allocation [32], but not the evolution of its sex allocation plasticity [31].

**Fig. 1:**
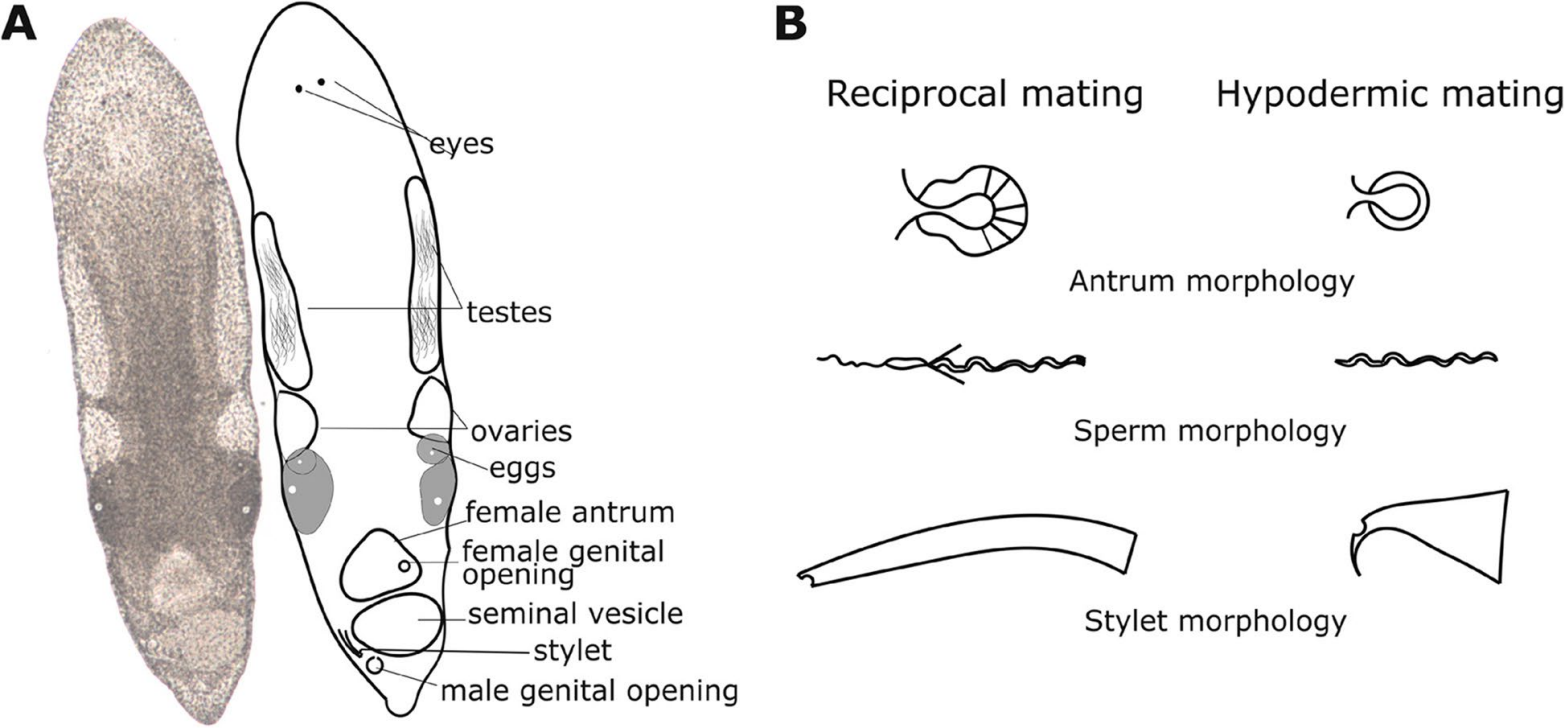
**A** Photograph and line drawing of an adult *Macrostomum cliftonense* (previously *M. cliftonensis*, see [34]), showing some of the components of the reproductive system to help understand the mating behaviour observations (total length ~ 1.2 mm). **B** Schematic drawings of the typical morphology of the female antrum (female reproductive organ), sperm and stylet (male intromittent organ) of *Macrostomum* species with the reciprocal mating syndrome (i.e. complex female antrum, complex sperm morphology and stylet with a blunt distal end) and the hypodermic mating syndrome (i.e. simple female antrum, simple sperm morphology and a needle-like stylet) [33]

Interestingly, *Macrostomum* species exhibit different combinations of reproductive morphological traits that are likely associated with the reciprocal mating and hypodermic insemination strategies (Fig. 1A,B). Indeed, a previous study [23] demonstrated an association between certain male and female reproductive traits (i.e. male and female genital morphology and sperm design) and the mating strategy in 16 *Macrostomum* species, naming the two alternative outcomes the reciprocal mating syndrome and the hypodermic mating syndrome. A more recent study [33] developed a more refined composite measure, called the inferred mating syndrome, which we use in this study. This measure is derived from the observation of additional components of the reproductive morphology (see “ Methods”), and was used to classify 145 *Macrostomum* species as exhibiting either the reciprocal inferred mating syndrome or the hypodermic inferred mating syndrome (Fig. 1B) [33]. Briefly, species were classified as exhibiting the reciprocal inferred mating syndrome if they had a blunt-tipped stylet (the male intromittent organ) with complex sperm featuring lateral bristles and received sperm in a typically complex female antrum. In contrast, species were classified as exhibiting the hypodermic inferred mating syndrome if allosperm was exclusively found hypodermically or if they had a simple female antrum (lacking thickening or a cellular valve) and sperm with reduced or absent bristles and a sharp stylet tip (see “ Methods” for details). The lateral bristles on the sperm in reciprocally mating species are hypothesised to represent a male persistence trait that allows the sperm to remain anchored in the female antrum and not be pulled out during the suck behaviour [22], whereas the thickened female antrum wall might prevent internal injury resulting from the male genitalia during mating. In contrast, the sharp needle-like stylet tip of hypodermically inseminating species likely allows sperm injection through the partner’s epidermis, while the simple sperm design presumably aids its movement through the partner’s body [23, 33].

Although sexual conflict has been studied in many organisms spanning different reproductive systems, studies on female resistance traits in hermaphrodites have been fewer, particularly in a phylogenetic context [23, 33, 35–39]. In our study, we examine the evolution of the suck behaviour, aiming to document reproductive behaviour in 64 *Macrostomum* species. We provide the first (quantitative) evidence that ejaculate is indeed removed during the suck behaviour, supporting the previously proposed functional hypothesis [22]. Using this extensive behavioural data set, we examine correlations between different aspects of the mating and suck behaviour, and between reproductive morphology and the behavioural mating strategies, while accounting for the phylogenetic interrelationships. If the suck behaviour has indeed evolved as a postcopulatory strategy, we predict positive correlations between the presence, duration and frequency of the mating behaviour and the suck behaviour. This could occur, e.g., if longer/frequent matings lead to more ejaculate being transferred, which would need longer/frequent sucks to remove the ejaculate (though note that we currently do not have evidence for a correlation between mating duration and the amount of ejaculate transferred in *Macrostomum*). We might also expect a trade-off between copulation duration and frequency, if species that spend a lot of time in copulation cannot copulate that often, e.g., due to ejaculate limitation or matings taking up a lot of the total time (so that fewer matings could be done over a period, i.e. an autocorrelation). Similarly, if the suck behaviour functions to remove ejaculate, we may expect a trade-off between suck duration and frequency, if shorter sucks require more frequent sucks to remove the ejaculate. Finally, we also expect the reproductive morphology to be a good proxy for inferring the behavioural mating strategy as a result of coevolution.

## Results

### Sperm deposition and removal during mating and suck behaviour in Macrostomum hamatum

**Fig. 2:**
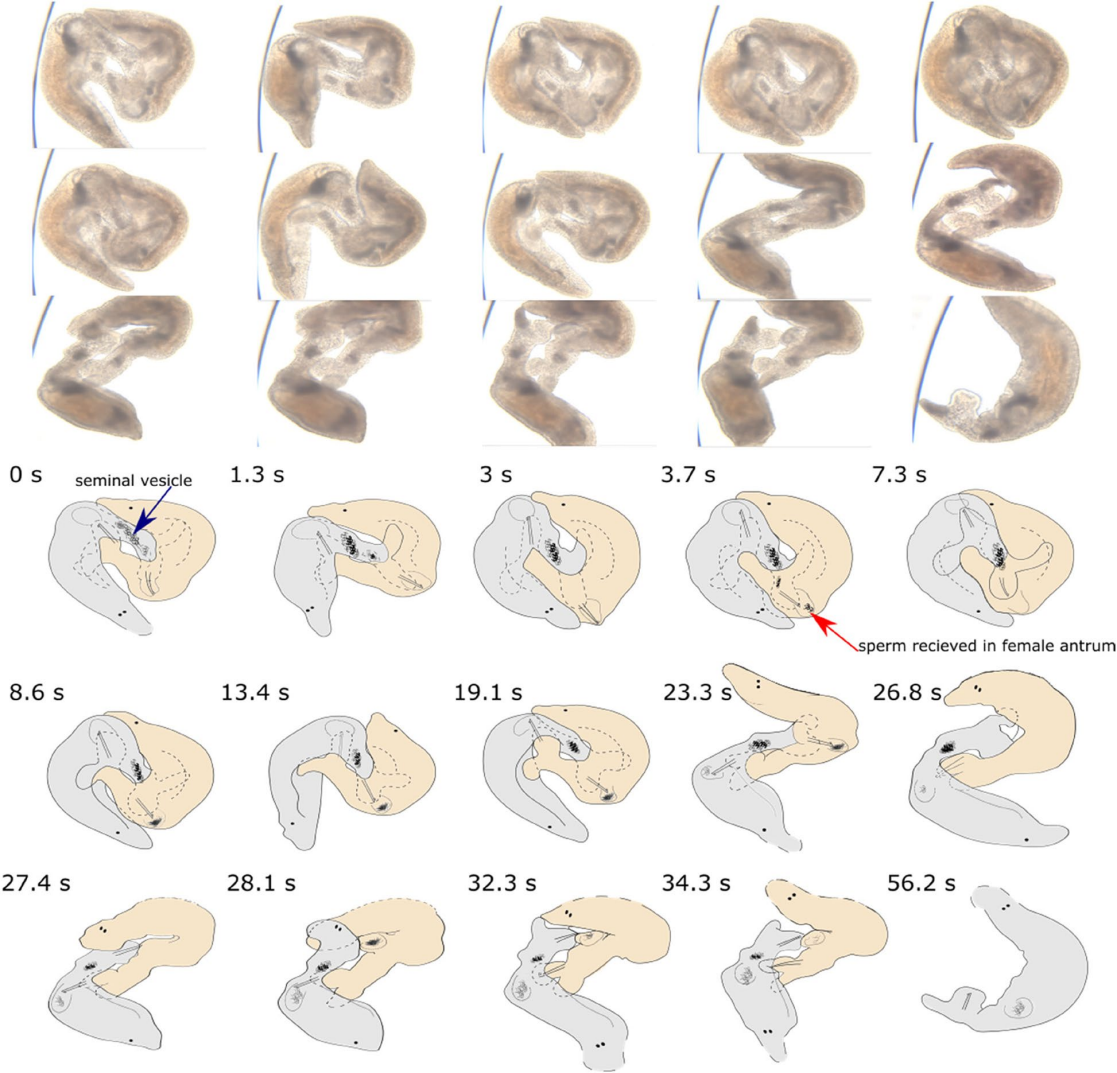
Reciprocal mating followed by a postcopulatory suck in *Macrostomum hamatum*, including ejaculate deposition by Grey (the worm on the left at 0 s) and its subsequent removal during the suck behaviour by Orange (the worm on the right at 0 s). Before transfer, the sperm is stored in the seminal vesicle of Grey (blue arrow in first frame), which is connected to its stylet. Ejaculate (dark mass indicated by red arrow) can be seen being deposited by Grey from the seminal vesicle starting from 3.7 s in the female antrum of Orange, followed by Orange pushing its female antrum region out (at 27.4 s) and sucking (note that Orange is also depositing ejaculate in Grey from 23.3 s). There is a visible reduction in the quantity of received ejaculate in the female antrum of Orange after the suck ends. Note that we call the frame from where we start describing the movie as *t* = 0 s, but the mating had already started before that timepoint. In some frames, parts of the worms are not visible on the video, and the presumed outlines are drawn using stippled lines. A high-resolution version is provided in Additional file 4: Figure S1A

We examined ejaculate removal during the suck behaviour in *M. hamatum*, using detailed close-up movies of field-collected specimens to visualise both sperm deposition and subsequent removal, to confirm the function of the postcopulatory suck behaviour. The general anatomy of the reproductive organs of *M. hamatum* is similar to that of many other reciprocally mating *Macrostomum* species (Fig. 1A,B). In the detailed movie of *M. hamatum*, the worms are already interlinked in the reciprocal copulatory position at the beginning of the clip (Fig. 2, Additional file 1: Movie S1), and we consider this as *t* = 0 s (hereafter we refer to the worm on the right as Orange and the worm on the left as Grey, respectively). At this timepoint “the tail plates touch each other ventrally in opposing directions, while the anterior ventral surface of each worm touches the posterior dorsal surface of the partner”, as previously described for the copulatory position in *M. lignano* [21]. Interestingly, in *M. hamatum* the copulatory position resembles a square with rounded corners, as opposed to *M. lignano*, where it is more circular. This may be due to a strikingly different position of the tail plate, which in *M. hamatum* stands at a 90° angle from the posterior body axis and appears to poke into the anterior ventral surface of the partner, leading to a dorsal bulge in both Orange and Grey.

Moreover, *M. hamatum* has a much more prominent erection (i.e. a translucent finger-like structure on the ventral tail plate, likely formed by the eversion of the muscular male antrum), which pokes into the posterior ventral surface of the partner in the region of the female genital opening (although it is unclear if the erection actually enters the partner). The stylet of Grey—while moving inside of the relatively stationary erection—then performs poking movements that are directed towards Orange’s female antrum, initially without any transfer of ejaculate. At *t* = 3–5 s, the stylet of Grey is seen repeatedly poking against the dorsal side of Orange’s female antrum wall, each time leading to a visible bulge on Orange’s dorsal side. In some of these frames one can see the sharp hook-shaped distal end of the stylet that is typical for *M. hamatum*. Eventually, Grey begins to deposit ejaculate (seen as a visible darkening of Orange’s female antrum lumen starting at about *t* = 5 s). During this process the seminal vesicle of Grey empties (as seen at the base of the erection, see also drawing in Fig. 2 at 0 s for location), while the female antrum of Orange fills up with ejaculate over the next ~ 21 s (see Fig. 2 from 3.7 s). Note that we here mainly focus on the sperm transfer from Grey to Orange, but in the meantime, Orange also pokes and eventually enters the female antrum of Grey (*t* = 16–20 s) and sperm is also transferred from Orange to Grey (between *t* = 21–27 s), although this is more difficult to follow in the movie.

**Fig. 3:**
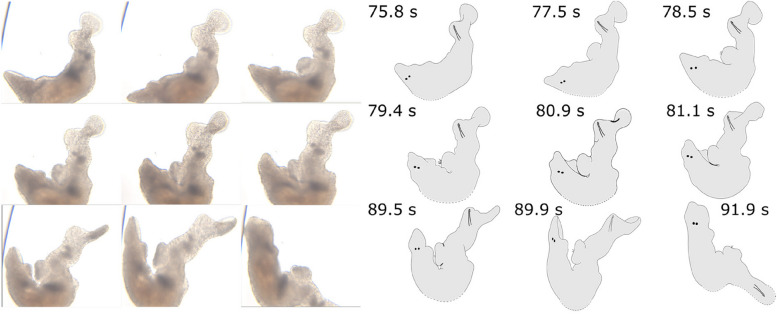
A postcopulatory suck following the reciprocal mating shown in Figure 2 (a continuation of the same movie), performed by Grey (i.e. the individual that had not yet sucked). Grey completely pushes out its female antrum region at *t* = 78 s (which leads to some sperm appearing near the female genital opening at *t* = 78.5 s), puts its pharynx over the female genital opening and then sucks out most of the previously deposited ejaculate over a period of 10 s (from *t* = 81 s). Moreover, some sperm can be seen sticking out of the female genital opening after the suck ends. In some frames, part of the worm is not visible on the video, and the presumed outline is thus drawn using stippled lines. A high-resolution version is provided in Additional file 4: Figure S1B

At *t* = 28 s, Orange pushes out its female antrum region, places its pharynx over its female genital opening, and then sucks. The received ejaculate can be seen leaving Orange’s female antrum (i.e. the visible darkening in the female antrum lumen moves towards the pharynx between *t* = 29–30 s). In total, the suck behaviour lasts for 7 s. Interestingly, during the suck the stylet of Grey remains anchored in Orange’s female genital opening (probably involving the abovementioned hook). At *t* = 52 s, the mating ends after a mating duration of ~ 64 s (recall that the worms were already in copula at *t* = 0 s). At *t* = 56 s, only Grey is in frame and the received ejaculate in its female antrum is clearly visible. It continues to have a small erection despite the mating being over. At *t* = 78 s, Grey pushes its female antrum region out and some sperm is ejected from the female antrum at *t* = 80 s, notably before the pharynx makes contact (Fig. 3, Additional file 1: Movie S1). At *t* = 81 s, Grey puts its pharynx over its female genital opening and then sucks for 10 s. After the suck, some sperm can still be seen sticking out of the female antrum (similar to *M. lignano*, [21]), especially at 92 s, but most of the ejaculate has been removed from the female antrum. The female antrum remains slightly everted and the erection somewhat visible until at least 108 s.

#### Quantification of ejaculate transfer and removal

**Fig. 4:**
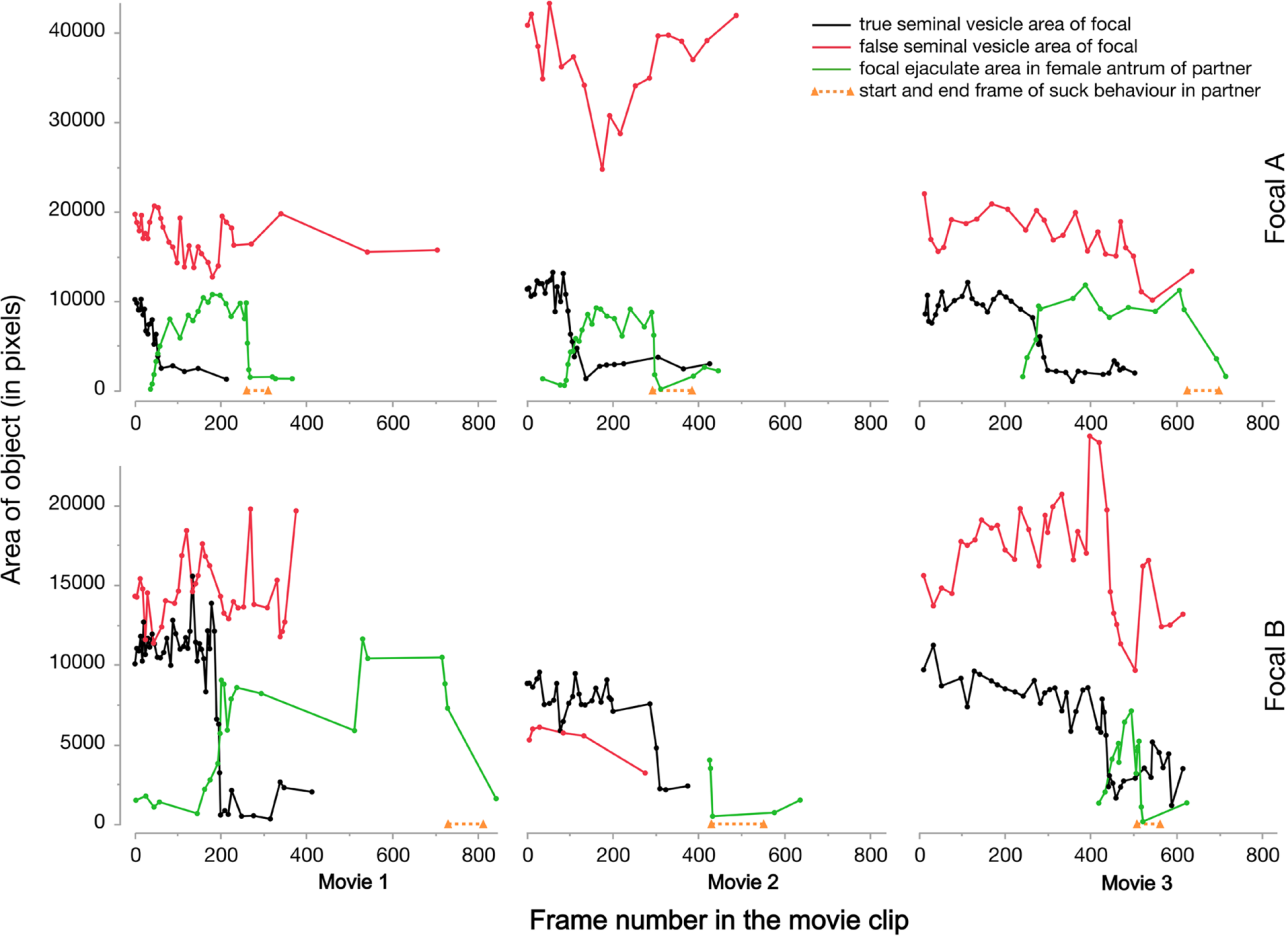
Quantification of ejaculate transfer and removal in three mating pairs observed in three movies of a group of field-collected Macrostomum hamatum. The lines connect neighbouring data points. The top panels consider one worm as the focal (Focal A) and the bottom panels consider its partner as the focal (Focal B). The movies are available in Additional file 1: Movie S1 (for high-quality movie, please see https://zenodo.org/records/6976430) and at https://zenodo.org/records/6957067

We quantified ejaculate transfer and removal in *M. hamatum* by analysing the movie frames and measuring changes in the seminal vesicle and female antrum using image analysis tools, to assess the dynamics of sperm deposition and subsequent removal during the suck behaviour. In all six focal worms, the true seminal vesicle area (black lines in Fig. 4) dropped at some stage during the mating and in all but one case one could simultaneously observe that the area of ejaculate in the female antrum of the partner increased (green lines in Fig. 4) (the female antrum could not be observed well in one partner, bottom middle, due to how it was positioned). Interestingly, the size of the drop in the true seminal vesicle was generally comparable to the size of the increase observed in the female antrum of the partner. A variable amount of time later, and observable in all cases, the area of the ejaculate in the female antrum dropped as they performed the suck behaviour (stippled orange line in Fig. 4), leading to most of the ejaculate being removed. The area of the false seminal vesicle did not change in an equally systematic way (red lines in Fig. 4), and it was very variable in overall size, likely reflecting the nature of an intermediate sperm storage structure. However, one could sometimes see that the drop in the true seminal vesicle area was accompanied by drops in the false seminal vesicle area, possibly due to sperm passing from the latter to the former.

### Evolution of the mating and suck behaviour across the genus Macrostomum

**Fig. 5:**
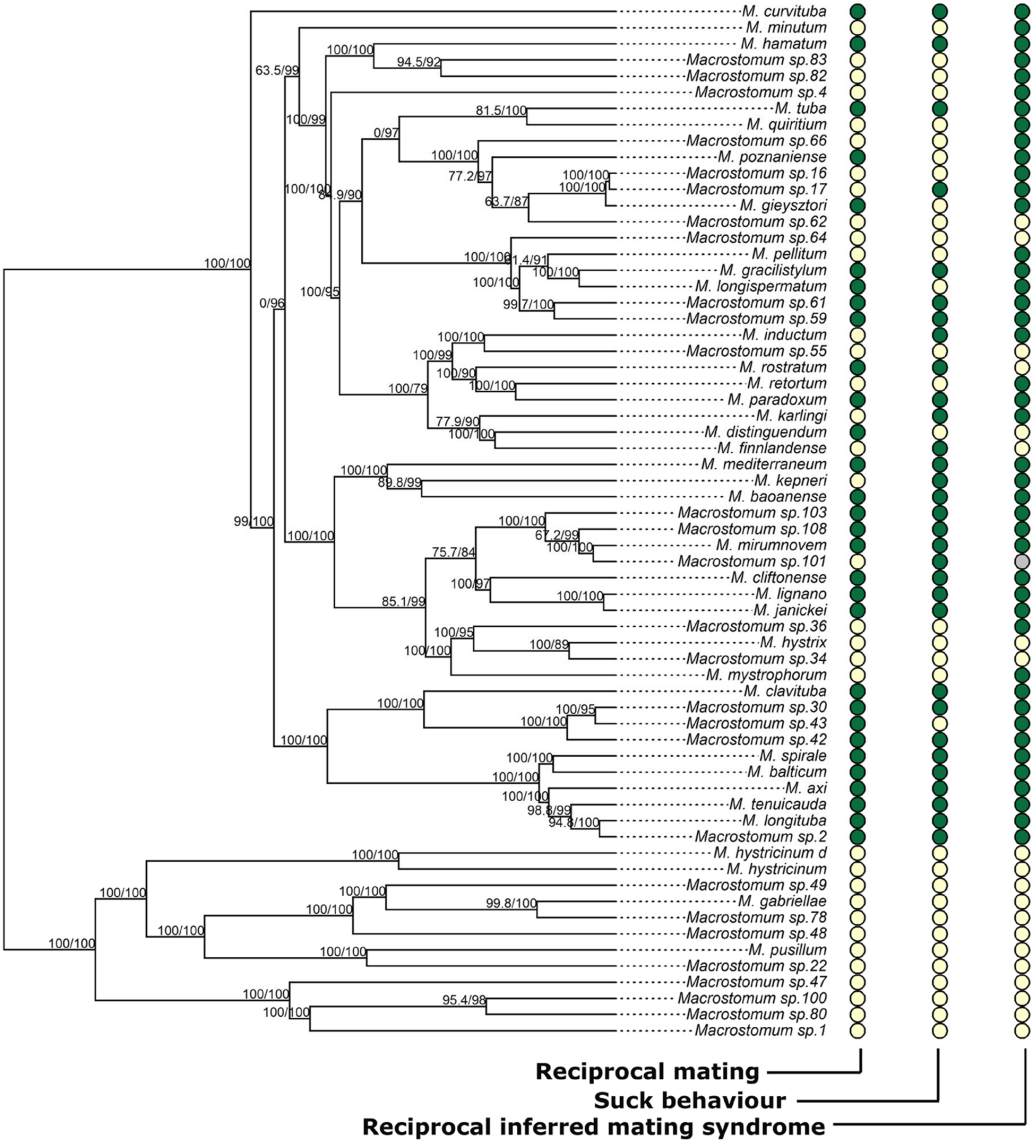
Presence (green) or absence (yellow) of reciprocal mating, the suck behaviour and the reciprocal inferred mating syndrome across the Macrostomum phylogeny (for a total of 64 Macrostomum species, see [40] for full phylogeny). Note that for the behaviourally inferred traits an absence may be due to a lack of sufficient data for observing the behaviour, and that for the reciprocal inferred mating syndrome the absence represents the hypodermic inferred mating syndrome (except for Macrostomum sp. 101, which showed an intermediate inferred mating syndrome, grey). Branch supports are indicated by ultrafast bootstrap (first number) and approximate likelihood ratio tests (second number), respectively [40]

We scored mating and suck behaviours across 64 *Macrostomum* species using frame-by-frame video analysis. We observed a total of 2796 worms across 64 *Macrostomum* species, with a mean of 44 worms observed and 76.7 h of observation time per species, for a total observation time of 4908 h. Of the 64 species, 30 species exhibited reciprocal mating behaviour, 31 species exhibited the suck behaviour and 25 species exhibited both the reciprocal mating and suck behaviour (Fig. 5).

#### Presence/absence of reciprocal mating and the suck behaviour

We tested for correlated evolution between reciprocal mating and the suck behaviour across *Macrostomum* species using a Bayesian discrete traits model, comparing dependent and independent evolutionary scenarios to assess whether the suck behaviour is associated with reciprocal mating. We found very strong support for the dependent model over the independent model of evolution for the correlation between the presence of reciprocal mating and the presence of the suck behaviour, with all three runs for each model providing highly consistent values (average marginal likelihood, independent = − 88.71, dependent = − 82.60, BF: 12.23; see also Additional file 4: Table S4a). This showed that the presence of reciprocal mating and the presence of the suck behaviour are strongly correlated. And the result was robust to observation time, since excluding the 6 species that were observed for < 21 h gave similar results (Additional file 4: Table S4A).

**Fig. 6:**
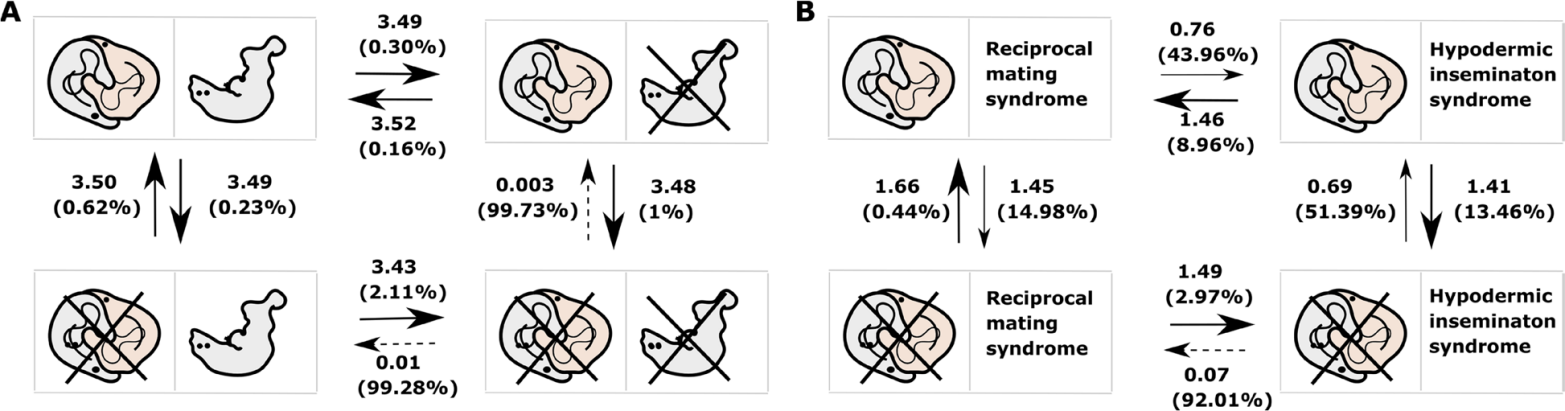
Correlated evolution of behavioural character states. The panels show the transition rates and the *Z* values (in brackets, expressed as %) for transitions between **A** the presence or absence of reciprocal mating and the suck behaviour (crossed out when absent) and **B** the presence or absence of reciprocal mating (crossed out when absent) and the inferred mating syndrome [40]. For the transition rates, the mean of the posterior distributions across all runs is given. The *Z* value can be understood as the percentage of times the transition rate was set to zero, among all the sampled parameters. The different arrows represent different probabilities of transitions between the states: high probability (strong black arrows, *Z* value < 15%), moderate probability (thin black arrows, *Z* value 20–55%) and low probability (dashed black arrows, *Z* value > 85%). The posterior distributions of the transition rate parameters are given in Additional file 4: Figure S2

The transitions from the absence of both the reciprocal mating and suck behaviour to the presence of either of these traits were found to be the most unlikely, as is evident from the low transition rates and the high *Z* values (Fig. 6A). Interestingly, the other transitions, including losing reciprocal mating or the suck behaviour from the state when they are both present, are all similarly likely. This contrast suggests that once both reciprocal mating and suck behaviour are lost or absent in a species, it is highly unlikely to regain either.

#### Presence/absence of reciprocal mating and the reciprocal inferred mating syndrome

We tested for an association between reciprocal mating and the reciprocal inferred mating syndrome (classified based on reproductive morphology) across *Macrostomum* species using a Bayesian discrete traits model, assessing whether mating behaviour and reproductive morphology coevolve. There was a clear correlation between the presence of reciprocal mating behaviour and the reciprocal inferred mating syndrome, as evident from the strong support for the dependent model over the independent model of evolution, with similar values for the three independent runs of each model (average marginal likelihood, independent = − 67.85, dependent = − 64.54, BF: 6.63; see also Additional file 4: Table S4b), suggesting that the reproductive morphology of a species can serve as a good proxy for its mating behaviour. And as before, our result was robust, as the reduced dataset gave us similar results (Additional file 4: Table S4B).

Transitions from the presence of reciprocal mating and the presence of the reciprocal inferred mating syndrome to the absence of either were moderately likely, while the converse transitions were very likely (Fig. 6B). Similarly, transitions from the absence of reciprocal mating and the absence of the reciprocal inferred mating syndrome to the presence of either were either unlikely or relatively unlikely, while the converse transitions were very likely. Together this suggests that there is a strong association between reciprocal mating behaviour and morphological traits characterising the reciprocal inferred mating syndrome (and between absence of reciprocal mating behaviour and the hypodermic inferred mating syndrome), such that species are attracted to these states and evolve away from states where the morphology and behaviour are mismatched.

#### Correlations between the frequency and the duration of mating behaviours

**Fig. 7:**
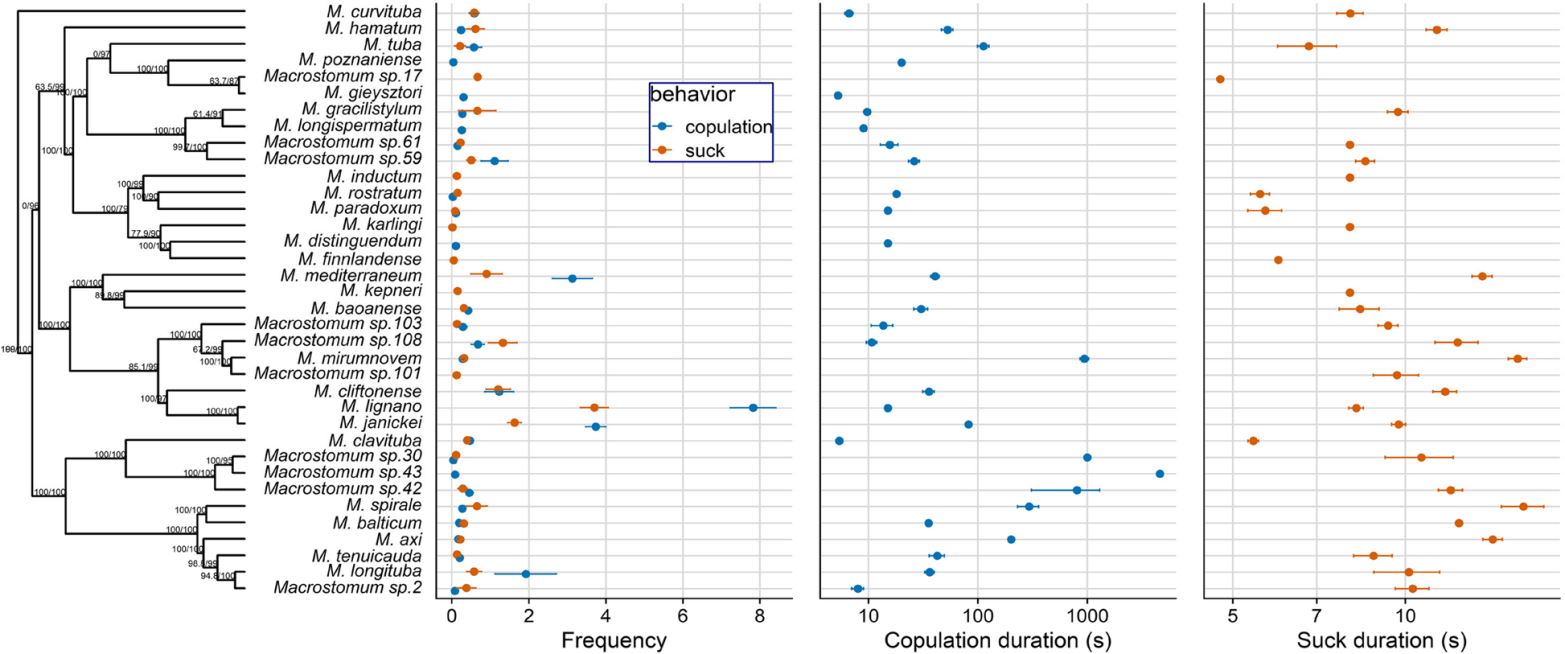
Trimmed phylogeny of the 36 *Macrostomum *species that showed reciprocal mating and/or the suck behaviour alongside data on means and standard errors of **A** reciprocal mating and suck frequency, **B** reciprocal mating duration (log-transformed) and **C** suck duration (log-transformed). Note that some species exhibited either only reciprocal mating or only the suck behaviour. Also note that for the species in which a behaviour had been observed in only 1 replicate, we report only that single value. The branch support values are indicated by ultrafast bootstrap (first number) and approximate likelihood ratio tests (second number), respectively [40]

Among the species that exhibited reciprocal mating (*n* = 30), the average mating frequency was 0.84 h^−1^ (range 0.02–7.82 h^−1^, Fig. 7A) and the average mating duration was 283.7 s (range 5.2–4609 s, Fig. 7B), with some sibling species showing fairly divergent values. Moreover, among the species that showed the suck behaviour (*n* = 31), the average suck behaviour frequency was 0.54 h^−1^ (range 0.01–3.7 h^−1^, Fig. 7A) and the average suck behaviour duration was 9.6 s (range 4.7–16.1 s, Fig. 7C).

**Fig. 8:**
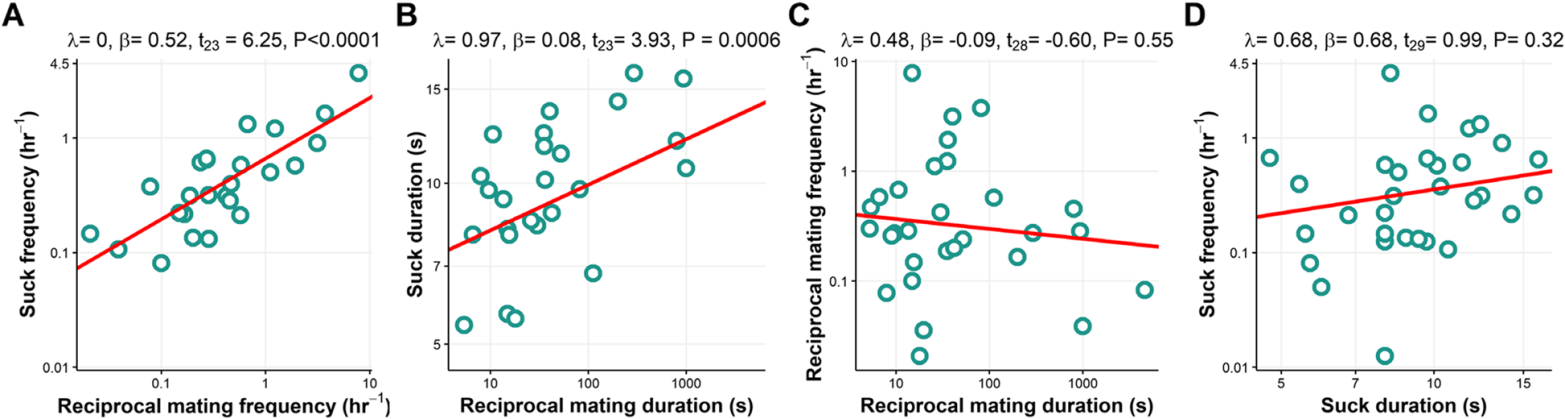
Relationships between **A** reciprocal mating frequency and suck frequency, **B** reciprocal mating duration and suck duration, **C** reciprocal mating duration and frequency and **D** suck duration and frequency for *Macrostomum* species. Note that **A–D** show values plotted on log-transformed axes with PGLS results

We used phylogenetic generalised least-squares (PGLS) regression to test for correlations between the frequency and duration of reciprocal mating and suck behaviours across *Macrostomum* species. In line with our predictions, we found significant positive relationships between both reciprocal mating frequency and suck behaviour frequency (Fig. 8A), and reciprocal mating duration and suck behaviour duration (Fig. 8B), while there was no significant relationship between reciprocal mating frequency and reciprocal mating duration (Fig. 8C), and suck behaviour frequency and suck behaviour duration (Fig. 8D). The reduced dataset also gave qualitatively similar results for all analysis (Additional file 4: Table S5).

## Discussion

Sexual conflict can give rise to antagonistic coevolution in all sexual systems [1, 41]. Here we documented the widespread occurrence of a putative female resistance trait, the suck behaviour, in > 30 species in the hermaphroditic flatworm genus *Macrostomum*. Moreover, the direct observation of ejaculate removal in *M. hamatum* corroborates the hypothesis that the suck behaviour functions as a female resistance trait to remove received ejaculate [21–23]. This interpretation is also supported by significant evolutionary correlations between different aspects of reciprocal mating and the suck behaviour. Finally, we showed that the reproductive morphology is a good proxy for the mating strategy of a species, presumably also as a result of coevolution. In the following, we discuss these findings in more detail.

### Sperm deposition and removal during mating and suck behaviour in Macrostomum hamatum

While multiple studies in *Macrostomum* have examined aspects of the suck behaviour [21, 23–27, 42], its involvement in removing received ejaculate components has so far only been hypothesised. Our detailed observations of mating interactions in *M. hamatum* provide the first direct evidence that ejaculate is indeed removed during this postcopulatory behaviour. However, as this species cannot currently be cultured in the laboratory, our sample size is limited (*n* = 6). Despite this, the consistency of our observations across multiple mating pairs strongly suggests that the suck behaviour functions in sperm removal. Future studies with a larger dataset, ideally in a species that can be maintained in the lab, could further quantify sperm removal and examine how factors such as mating status, partner identity, or size differences—known to influence suck behaviour in *M. lignano* [25, 26]—affect its occurrence. Interestingly, compared to *M. lignano* [21], *M. hamatum* has a more rectangular mating posture (possibly due to the angular position of the tail plate), a larger erection around the stylet, and the worms prominently evert the female antrum just before the suck behaviour, likely as a result of muscular contractions. This could result from differences in the female antrum morphology: while *M. hamatum* has a strong musculature and an inner second chamber connecting to the main female antrum [43], *M. lignano* has a somewhat simpler female antrum with a single chamber [22, 44]. Similarly, the prominent erection of the male antrum could result from a muscular morphology that is similar to the muscular cirrus seen in species of the sister genus, *Psammomacrostomum* [45, 46]. The combination of a rather prominent female antrum and the relatively transparent specimens may have helped us visualise the function of the suck behaviour better in *M. hamatum* than in other *Macrostomum* species observed to date.

We showed quantitatively that a reduction in the amount of ejaculate in the true seminal vesicle of a focal worm is accompanied by an increase in the amount of ejaculate present in the partner’s female antrum, and that the suck behaviour is then accompanied by a reduction in the amount of ejaculate in the female antrum (Fig. 4). Together this suggests that the ejaculate transferred during a copulation is subsequently removed during the postcopulatory suck behaviour. While we see ejaculate being removed during the suck behaviour, we cannot clearly determine whether it is ingested. Although sperm digestion is widespread in hermaphrodites [1, 47–50], it usually occurs inside an organ connected to the individual’s reproductive system, unlike in the case of the suck behaviour. To our knowledge, there have been only two earlier reports of sperm being orally taken up in hermaphrodites, one in the arrow worm *Spadella cephaloptera* [51] and the other in the leech *Placobdella parasitica* [52]. Thus, the suck behaviour seems to be a novel trait, which to date has only been observed in species of the Macrostomidae (including one member of the sister genus *Psammomacrostomum*; P. Singh, pers. obs.). The suck behaviour may work better in species that have separate male and female genital openings—as is the case in *Macrostomum*—although there is also the possibility of additional internal constrictions and/or sphincters in species with common genital openings [45, 53], which might still permit sucking out from only the female ducts. Similar to the suck behaviour, the consumption of spermatophores after mating occurs in multiple species, including females of the ladybird beetle, *Adalia bipunctata* [54]. Moreover, there is also sperm dumping in many separate-sexed species, in which the female physically ejects received sperm from her reproductive tract, and this, at least in some cases, is thought to be a mechanism of cryptic female choice [55–57]. If the suck behaviour also functioned in cryptic female choice, we might expect individuals to remove or retain sperm of certain partners more frequently (e.g. [58]). This has also been observed in *M. lignano*, where the propensity of the recipient to exhibit suck behaviour is affected by the mating status [25] and the genotype of its partners [26]. However, it is difficult to ascertain whether the suck implies an active choice by the recipient or whether it is sometimes prevented as a result of a manipulation by the donor [27]. Another possibility could be that the suck behaviour is induced by the donor to displace previously transferred rival sperm, or for proper attachment of the donated sperm in later matings. From a previous study, we know that not all matings lead to sperm transfer in *M. lignano* [59], and so some matings could potentially serve purposes other than sperm transfer. While reciprocal mating behaviour is well documented, and offspring production is typically observed in both individuals in multiple matings between virgin worms, direct evidence of sperm transfer being reciprocal in single matings in *Macrostomum* is still lacking. Our study provides a detailed account of the reciprocal transfer and deposition of sperm by both mating individuals during a reciprocal mating in *Macrostomum* (but see [60] which documents mating and unilateral sperm transfer in *M. salinum*, now considered to be *M. romanicum*).

### Evolution of the mating and suck behaviour across the genus Macrostomum

We found a significant evolutionary correlation between the presence of reciprocal mating and the suck behaviour (Fig. 6A). In reciprocally mating species, ejaculate is deposited in the female antrum allowing its removal during the suck behaviour, while in hypodermically inseminating species, sperm is injected potentially anywhere in the body [23, 33]. Given that the function of the suck behaviour indeed appears to be the removal of ejaculate, we do not expect to see the suck behaviour in hypodermically inseminating species. Performing a suck at a site of hypodermic insemination might not permit effective ejaculate removal (particularly also given the abovementioned active participation of the female antrum musculature), but instead would more likely lead to additional tissue damage. Interestingly, the transition rates showed that while it is unlikely for a species that lacks both the reciprocal mating and suck behaviour to gain either of these traits, the loss of either reciprocal mating or the suck behaviour was estimated as being more likely. These transitions could represent transitional steps towards hypodermic insemination, which might arise as a means to bypass the female control and allow access to the eggs [1, 33]. Moreover, this interpretation is also supported by the finding that there are multiple origins of hypodermic insemination in the genus *Macrostomum* [31, 33]*.* There are at least nine independent shifts from reciprocal mating to hypodermic insemination in *Macrostomum*, while no transition is observed in the converse direction [33].

However, it is important to point out that some of these findings could also have resulted from a lack of observations of either the reciprocal mating and/or suck behaviour (despite being present in a species), leading to an overestimation of these transition rates. Specifically, there were six species that showed only the reciprocal mating behaviour and five species that showed only the suck behaviour (Additional file 4: Table S1). These mismatches usually appeared in species for which we had comparatively few observation hours (for more detail see Additional file 4: Figure S3), suggesting that additional observations could help to further ascertain the actual presence/absence of reciprocal mating or the suck behaviour, respectively. Moreover, mismatches could result from a species not exhibiting some behaviours under our laboratory conditions, or they might indicate that a species indeed lacks a behaviour. In addition, if a species mates only rarely, individuals might be less inclined to remove the sperm they receive, and in our study, the species that showed reciprocal mating but did not suck, had low or intermediate mating frequencies (see *Macrostomum* sp. 43, *Macrostomum* sp. 67, *M. distinguendum*,* M. gieysztori*, and *M. poznaniense* in Fig. 7A). Alternatively, species might actually lack reciprocal mating, but losing a resistance trait like the suck behaviour might take longer, particularly if the suck behaviour does not impose costs on the fecundity. Moreover, the suck behaviour could have additional functions, such as possibly removing egg material that remains in the female antrum after egg laying, with a previous study in *M. lignano* showing that 24% of the observed suck behaviours occurred independently of a copulation [21]. Species are predicted to lose defensive or resistance traits only after the persistence traits have become substantially less harmful, leading to a time lag [2]. A study on the seed beetle, *Callosobruchus maculatus*, showed that, while large males evolved relatively reduced length of genital spines under monogamy, there was no detectable evolution in female genitalia within the same time period [61]. And finally, since the worms we observed may often have mated before we placed them into the mating chambers, some of the observed sucks might have occurred in response to unobserved earlier matings, since sucks do not only occur immediately after mating [21].

The significant evolutionary correlation between the presence of reciprocal mating and the purely morphologically derived reciprocal inferred mating syndrome (Fig. 6b) confirms previous findings [23]. It shows that persistence and resistance are not generally limited to single traits, but are often composite suites of behavioural, morphological, and physiological traits acting together [3]. For example, the thickened female antrum wall and the suck behaviour might be different components of female resistance. While the former might prevent injury resulting from the male genitalia when mating reciprocally, the suck behaviour serves to remove unwanted ejaculate received during mating. Similar adaptations of the female reproductive tract are also seen in the seed beetle *C. maculatus*, where a thicker female tract lining serves as a resistance trait against harm by male genitalia [62]. Moreover, resistance and persistence traits can also occur at the proteomic level. A study in *M. lignano* identified two seminal fluid transcripts, experimental knock-down of which caused mating partners to suck more often [27]. This suggests that the seminal fluid proteins derived from these transcripts might be counter adaptations by the donor to prevent the suck behaviour by the recipient. And effects of seminal fluids on the partner’s reproductive behaviour have also been documented in the great pond snail, *Lymnaea stagnalis*. In this species, individuals that received ejaculate components, such as accessory gland proteins, had a greater propensity to exhibit avoidance behaviours [63], which could potentially deter insemination by sperm donors, and they also showed a reduced amount of ejaculate transferred in subsequent matings [64].

In our dataset, there was one species each that exhibited the hypodermic inferred mating syndrome morphology and showed both reciprocal mating and the suck behaviour (*M. rostratum*), only reciprocal mating (*M. distinguendum*), or only the suck behaviour (*M. finlandense*) (Additional file 4: Table S1). Interestingly, the three species represent at least two, but possibly three, of the abovementioned multiple independent origins of the hypodermic inferred mating syndrome [33]. Conversely, there were 12 species that exhibited the reciprocal inferred mating syndrome, but in which neither reciprocal mating, nor the suck behaviour was observed. As above, this mismatch occurred mainly in species for which we had relatively few observation hours (Additional file 4: Figure S3), suggesting that, if these species have a low mating frequency, then more observation time may be needed to avoid falsely inferring the absence of the mating and suck behaviour. And finally, for many of the species that showed the hypodermic inferred mating syndrome we had considerable amounts of observation hours (Additional file 4: Figure S3), so that it seems unlikely that the absence of mating and suck behaviour observations in these species were due to a lack of effort.

*Macrostomum* species showed large interspecific variation in behaviour, with a nearly 900-fold variation in mating duration, a threefold variation in the suck duration, and a nearly 400-fold variation in the mating and suck frequency across the genus (Fig. 7). Remarkably, despite this extensive interspecific variation in behavioural traits, we see clear correlations between both the mating and suck duration, as well as the mating and suck frequency, suggesting that the mating and suck behaviour have coevolved. If a longer mating duration or more frequent mating implies more sperm transfer, then we expect selection for a longer suck duration and/or a more frequent suck behaviour (particularly if ejaculate receipt is associated with fitness costs). In some species, at least, a longer mating duration does imply more ejaculate transferred [65] and is often used as a proxy for ejaculate size [66]. Alternatively, such a correlation could also emerge as a result of variation in genital complexity, e.g. if it takes longer to insert and remove more complex male genitalia, and to suck out ejaculate from more complex female antra. Interestingly in *Macrostomum*, male and female genital complexity are indeed correlated [33]. Moreover, a positive correlation between reciprocal mating and suck could also appear, if some species do not do well under our laboratory conditions, leading to an overall low behaviour frequency. Note, however, that we confirmed that the individuals we used for mating movies were adults with visible testes and ovaries, and we also established the robustness of the observed correlations by excluding species in which mating or suck had only been observed in one replicate (Additional file 4: Table S5). We did not find any correlations between the frequency and duration for either reciprocal mating or suck. While we might have expected mating duration to trade off with mating frequency, mating duration only made up a relatively small percentage of total time, potentially posing no trade-off. Similarly, if sucking is not very costly, the suck behaviour duration and frequency may not trade-off and could even be positively correlated, since both help to remove ejaculate. However, it remains possible that such trade-offs exist at the individual level within species (e.g. due to resource allocation constraints), even if they are absent at the between-species level.

Finally, mating frequency (and possibly mating duration) could be positively correlated with allocation towards the male function (e.g. testes). Indeed, studies in *Macrostomum* have shown interspecific variation in sex allocation towards the male and female functions, such as testes and ovaries [30–32]. This interspecific variation could potentially relate to the mating behaviour, as we can expect species that have a longer mating duration or higher mating frequency to have larger testes, if longer and/or more frequent mating implies that more sperm are transferred [67]. Mating duration could also correlate with the complexity of genitalia, such that more complex genitalia might require longer mating duration [68]. While we found a significant correlation between reciprocal mating and reproductive morphology, our analysis treated this as a binary classification based on the inferred mating syndrome. Future studies should investigate whether specific components—such as stylet shape, sperm bristles, or female antrum thickness—contribute differentially to these correlations and whether certain traits evolve more independently than others within the mating syndromes of *Macrostomum*.

## Conclusions

Our study provides direct observational evidence for ejaculate removal during the postcopulatory suck behaviour in the species *M. hamatum*, compelling support for the coevolution between the reciprocal mating and suck behaviour, and detailed information in a phylogenetic context on the occurrence and interspecific variation of the suck behaviour. Moreover, we show that reproductive morphology can be a good proxy to infer the behavioural mating strategy. Taken together, our study shows the presence of a postcopulatory female behavioural resistance trait that co-evolves with mating strategy and allows manipulation of received ejaculate in a simultaneously hermaphroditic sexual system. Thus, our study adds to the repertoire of information on traits involved in sexual conflict in *Macrostomum* genus and demonstrates the genus as an excellent model system for understanding sexually antagonistic coevolution by allowing us to examine the evolution of diverse female resistance and male persistence traits, spanning behavioural and morphological traits, simultaneously.

## Methods

### Study organisms

Species in the genus *Macrostomum* are small (~ 0.3 to 3.0 mm body length) aquatic free-living flatworms that are highly transparent, permitting detailed observations of internal structures (for the general morphology see Fig. 1A,B). The sperm and eggs are produced in the paired testes and paired ovaries, respectively, with studies documenting inter- and intra-specific variation in both testis and ovary size across the genus [30–32]. The female antrum is located anterior to the male antrum, connected to the outside, respectively, via a female genital opening (also female genital pore or vagina) and the male genital opening (also male genital pore). The stylet (male intromittent organ) resides within the male antrum and it is proximately connected via the vesicula granulorum (not shown) to the seminal vesicle, which contains sperm to be transferred during mating. In both reciprocally mating and hypodermically inseminating species, the female antrum serves as the egg-laying organ, while in reciprocally mating species it additionally serves to receive the stylet during mating and as the sperm-storage organ [22, 23].

We obtained multiple specimens for a large number of *Macrostomum* species, collected from a range of locations and habitats, using a variety of extraction techniques, which we report on in more detail as part of separate studies on the phylogenetic interrelationships [40] and reproductive character evolution in this genus [33]. Briefly, most specimens were sampled directly from natural field sites, while some were sampled from artificial ponds, or from aquaria containing other study organisms, and they were generally observed within a few days of collection. Other specimens were obtained from short- and long-term laboratory cultures maintained either by our group or by colleagues. Note that some species have been formally named now, see Additional file 4: Table S1 and [69].

Following [33] that used the reproductive morphology to infer the mating syndrome, 38 of a total of 64 *Macrostomum* species included in the current study were classified as exhibiting the reciprocal inferred mating syndrome, because they had a blunt tip of the stylet, and of these all but one had received sperm in the female antrum. This exception was *Macrostomum* sp. 30, which was initially recorded (based on one specimen from Noto, Ishikawa Prefecture, Japan in 2008) as having no observed received sperm in the antrum [33]. However, our analysis of additional worms collected from Iriomote, Okinawa Prefecture, Japan in 2019 revealed received sperm in two out of three specimens examined (see https://zenodo.org/records/14882469 for information on sample location, specimen images, and molecular data). Additionally, upon closer inspection, we found that the specimen from Noto also had received sperm. These observations further support the classification of *Macrostomum* sp. 30 within the reciprocal inferred mating syndrome. A further 6 species with a sharp stylet were also classified as exhibiting the reciprocal inferred mating syndrome because they had complex sperm with lateral bristles and we observed sperm in the female antrum (Fig. 5). In contrast, 15 species were classified as exhibiting the hypodermic inferred mating syndrome because allosperm was exclusively found hypodermically. An additional 4 species without observation of received sperm were classified as exhibiting the hypodermic inferred mating syndrome because they had a simple female antrum (no thickening of the female antrum wall and no visible cellular valve), a sperm design with reduced or absent bristles and a sharp stylet tip. Finally, one species (*Macrostomum sp*. 101) was classified as intermediate because received sperm was observed both in the female antrum and within the tissue (Fig. 5).

### Observation methodology

We aimed at documenting the mating behaviour of all 64 *Macrostomum* species, by placing the worms in mating chambers [21]. A mating chamber consisted of the worms being placed between two microscope slides in small drops (i.e. either freshwater or water with different salinity, depending on the collection habitat), with a variable number of spacers separating the slides (see below), and sealed with pure white Vaseline (note that we generally also placed 4–6 empty drops around, to reduce evaporation). We adjusted the spacer number and drop volume depending on the size and number of worms in a drop, respectively. Usually, for a pair of worms of the size of *M. lignano* (~ 1.5 mm body length), we used 2 spacers (each spacer being ~ 105 μm) and a drop size of ~ 3 μl. Movies were recorded when specimens were available and, therefore, across several sampling campaigns. Consequently, the recording setups differed (macro lenses, cameras or lighting conditions). However, a previous detailed study of two *Macrostomum* species showed that these minor setup differences are unlikely to bias the observations [42]. Usually, the movies were recorded in QuickTime Format using BTV Pro (http://www.bensoftware.com/) at 1 frame s^−1^. But for some species we also generated detailed close-up movies, where worms were manually tracked at higher magnifications under a compound microscope and filmed at higher frame rates (see next section). All worms were visually checked for sexual maturity (defined as having visible gonads or eggs) either before or after filming.

### Detailed observation of mating and suck behaviour in Macrostomum hamatum

While earlier work documented sperm sticking out of the female antrum after the suck behaviour in *M. lignano* [21, 23], direct observations of ejaculate removal have not been reported to date. Here we could document ejaculate removal in detailed close-up movies of *M. hamatum*, possibly since field-collected specimens of this species appeared to be more transparent than other species. This allowed us to clearly visualise the deposition and subsequent removal of ejaculate during the mating and suck behaviour, respectively. Specifically, we examine the mating behaviour of *M. hamatum* (collected on 27 July 2017 directly in front of the Tvärminne Zoological Station, Finland; N 59.84452, E 23.24986) in a detailed close-up movie (Additional file 1: Movie S1). Note that while we describe and illustrate only one such instance (in an extract from a longer movie of three *M. hamatum* individuals), we also observed ejaculate removal in other detailed close-up movies of *M. hamatum* that we also deposit (https://zenodo.org/records/6957067), and these observations corroborated our finding as described here (see below and Fig. 4).

#### Quantification of ejaculate transfer and removal

We aimed to quantify the amount of ejaculate transferred from a focal worm to its partner, as well as the subsequent removal of ejaculate as that partner performs the suck behaviour. Movies showing reciprocal copulatory interactions in *Macrostomum hamatum* were converted to TIF-stacks using the Import > Movie (FFMPEG)… function of Fiji (running ImageJ2 v2.3.0/1.53). Uninformative portions were sometimes trimmed to reduce file size. This included the movie discussed earlier (see also Figs. 2 and 3) and two further movies obtained from the same group of field-collected worms. These TIF-stacks were then imported into the ObjectJ plugin (v1.05n), allowing placement of non-invasive overlays to measure objects on movie frames. Three objects were defined in ObjectJ and measured in focal A, namely the area of its true seminal vesicle (recognisable by the muscular sheath), the area of its false seminal vesicle (situated more anterior and lacking a muscular sheath) and the area the focal’s ejaculate inside the female antrum of the partner. The equivalent data were subsequently collected when the partner was considered the focal (focal B). Given the constant movement of the worms and the limited focal depth at this high magnification, the different objects were not equally well visible in all frames of a movie, but constant adjustment of the focal plane of the microscope allowed us to identify and measure each object many times over the course of the mating interaction. Moreover, the projection area of an object of course only approximates the volume of the structure of interest, likely also explaining some of the observed variation. For each focal worm, we first measured the true seminal vesicle across all relevant frames, and then moved on to measure the false seminal vesicle, and then the ejaculate in the female antrum of the partner. Each time we chose frames that showed the object of interest optimally without reference to the other objects, making observations of the different objects more independent. This yielded an average of 34.2 (range 20–48), 25.0 (range 6–35) and 18.0 (range 5–26) measurements for these three objects, respectively. Moreover, for each focal we noted the start and the end frame of the suck behaviour of its partner (see below for the criteria used to score the start and end).

### Scoring of mating and suck behaviour across species

We scored the mating behaviours from the mating movies by visual frame-by-frame analysis (Additional file 4: Table S1). A reciprocal mating was scored when the tail plates of two worms were in ventral contact and intertwined, such that the female antrum was accessible to the partner’s stylet and vice versa, which would allow reciprocal transfer of ejaculate. In most species, the copulatory posture is accompanied by the pair being tightly interlinked (like two interlocking Gs, see Fig. 2), and thus similar to the mating behaviour originally described for *M. lignano* [21]. Note that in some species the mating posture can deviate from that observed in *M. lignano* (see Additional file 4: Table S2), such as, for example, in *Macrostomum* sp. 57 and *Macrostomum* sp. 61 (Additional file 2: Movie S2A,B). The mating duration was measured from the frame when the tail plates were in ventral contact (and usually tightly intertwined), to the frame where the tail plates were no longer attached to each other. We defined behaviours as matings only if the pair was in the above-described posture for at least 3 s. The suck behaviour duration was measured starting from the frame when an individual placed its pharynx over its female genital opening, up to the frame where the pharynx disengaged. Note that in some cases, individuals do not lie on their side while sucking (as generally seen in *M. lignano*), which can sometimes make it more difficult to observe the suck behaviour. For each replicate drop, we divided the total number of mating and suck behaviours by the number of worms and the movie duration to obtain a standardised value. We then averaged the frequency and duration estimates across all replicate drops for each species to obtain the species estimates of the respective behaviours.

While we also invested significant effort into observing hypodermic insemination (see Additional file 4: Table S1 and Results), we only saw some rare behavioural instances in a few species that could possibly represent cases of hypodermic insemination, such as, for example, in *Macrostomum* sp. 1 and *M. gabriellae* (Additional file 3: Movie S3A,B). Possible reasons for not observing hypodermic insemination could be that in many species such matings occur very rapidly or that they mate less frequently, possibly since they try to avoid sperm receipt [9, 70]. Given that we could not confirm the presence of hypodermic insemination, we scored species either as having reciprocal mating being present (when it was observed) or absent (when it was not observed) (Fig. 5). Note, however, that the absence of observations of reciprocal mating does not necessarily imply the presence of hypodermic insemination. Instead, it could also result from a reciprocally mating species not mating under laboratory conditions, from an overall low mating frequency of a species, and/or from a species being selfing (though note that the only known reciprocally mating species capable of selfing, *M. mirumnovem*, readily mated when it had access to partners [30];). The absence of reciprocal mating thus only signifies that no reciprocal mating was observed. Similarly, while the presence of the suck behaviour can clearly be identified in many species, the absence of observations of the suck behaviour does not necessarily mean that a species never shows this behaviour.

### Evolution of the mating and suck behaviour across the genus Macrostomum

To perform phylogenetic comparative analyses, we used a trimmed version of a recently published ultrametric large-scale phylogeny of the genus *Macrostomum* (i.e. the C-IQ-TREE phylogeny of [40]). This phylogeny is based on an amino acid alignment of 385 genes from 98 species, supplemented with Sanger sequences from a *28S rRNA* fragment, which allowed the addition of a further 47 species, and calculated using a maximum likelihood approach [40], covering all the species we included in the current study. Specifically, we determined (1) whether the presence/absence of reciprocal mating is correlated with the presence/absence of the suck behaviour, (2) whether the presence/absence of reciprocal mating is correlated to the presence/absence of the reciprocal inferred mating syndrome (which describes a combination of morphological traits associated with the mating syndrome; see Fig. 1) and (3) whether there are correlations between the frequency and the duration of the reciprocal mating and suck behaviours among the species that show these behaviours.

#### Presence/absence of reciprocal mating and the suck behaviour

We used the DISCRETE model in BayesTraits V.3.0.1 to test for correlated evolution between reciprocal mating and the suck behaviour (both scored as present/absent), using the Reversible Jump Markov Chain Monte Carlo (RJ MCMC) approach [71–73]. Specifically, we compared the marginal likelihood of a dependent model, in which the presence of suck behaviour depends on the presence of reciprocal mating, to an independent model, in which the suck behaviour and reciprocal mating evolve independently. Each RJ MCMC chain was run for twelve million iterations and the first one million iterations were discarded as burn-in, after which the chain was sampled every 1000th iteration. We used a gamma hyperprior (gamma 0 1 0 1) and placed 1000 stepping stones (with each iterating 10,000 times) to obtain the marginal likelihood values for the models. We ran three separate chains each for the dependent and independent model to check for the stability of the likelihood values and convergence. Using the R package coda [74], we confirmed that the chains had converged [75, 76] and that the Effective Sample Size was > 200 for all parameters. In addition, we also confirmed that the acceptance rate was between 20 and 40% [72]. We compared the alternative models with the Log Bayes Factor (BF), using the convention that BF values > 2 are considered as positive support for the best-fit model, while values between 5–10 and > 10 are considered as strong and very strong support for the model, respectively [72]. To examine the robustness, we repeated the analysis for a reduced dataset, by excluding six species that had in total been observed for < 21 h (~ 10% quantile). For the dependent models of the full dataset, we estimated the transition rates among the different trait states by calculating *Z* values. This value can be understood as the percentage of times a transition rate was set to zero, with a high value thus indicating that the transition between two states is unlikely. We expect a correlation between the presence of reciprocal mating and the presence of the suck behaviour, which would corroborate that the suck behaviour indeed is a postcopulatory behaviour that is linked to reciprocal mating, rather than possibly serving a function that is also present in species with hypodermic insemination.

#### Presence/absence of reciprocal mating and the reciprocal inferred mating syndrome

We checked for an association between reciprocal mating (scored as present/absent) and the inferred mating syndrome (scored as reciprocal/hypodermic), using the DISCRETE model in BayesTraits V.3.0.1 (as above). One of the species, *Macrostomum* sp. 101, had a morphology that was scored intermediate between reciprocal and hypodermic [33], but since the discrete method in BayesTraits only allows binary trait states, we excluded this species from this analysis. We expect a correlation between the presence of reciprocal mating behaviour and the reciprocal inferred mating syndrome, which could indicate that behaviour and morphology coevolve.

#### Correlations between the frequency and the duration of mating behaviours

In preparation for phylogenetic correlation analyses, we estimated the phylogenetic signal for the continuous traits (i.e. the duration and frequency of both the reciprocal mating and the suck behaviour; log-transformed for all the analyses) using Pagel’s *λ* [77, 78]. A *λ* value of 1 indicates a strong phylogenetic signal, while a value around 0 indicates no/low phylogenetic signal [77]. We found a phylogenetic signal that was significantly different from 0 for the suck behaviour frequency (*λ* = 0.67, *P* = 0.02), the suck behaviour duration (*λ* = 0.76, *P* = 0.005) and the reciprocal mating frequency (*λ* = 0.50, *P* = 0.05), but only marginally so for the mating duration (*λ* = 0.46, *P* = 0.06). For each trait, we then fitted four different models of trait evolution, i.e. Brownian motion, Ornstein–Uhlenbeck, Early-burst and Lambda models [79]. We found that the Lambda model had the lowest sample-size corrected Akaike Information Criterion (AICc) weights (ω_ι_) (Additional file 4: Table S3), and this model was hence chosen for further PGLS analysis.

For the species that exhibited both reciprocal mating and the suck behaviour, we then investigated if there was a correlation between the frequency and duration of the reciprocal mating and suck behaviours, using phylogenetic generalised least-squares (PGLS) regression implemented in the *caper* package version 1.0.1 [80]. PGLS accounts for the non-independence of the data by incorporating the phylogenetic relationships between species into the error structure of the model. For each analysis using the frequency and duration of the reciprocal mating and suck behaviours, the phylogenetic signal (Pagel’s *λ*) was estimated using the maximum likelihood approach. We examined the residuals of each model for normality and homogeneity [81]. Additionally, we scrutinised for influential cases (species) in each PGLS model, by excluding one species at a time from the data and rerunning the analysis, and comparing the results obtained with the results for the entire dataset [81]. And finally, we evaluated the robustness of our results by repeating the PGLS for a reduced dataset, which excluded five species in which mating or suck behaviour had only been observed in one replicate (note that this reduced dataset is different from the reduced dataset used in the above BayesTraits analysis).

We performed our analysis in R, version 3.6.1 [82].

## Supplementary Information

Additional file 1. Movie S1. Movie of the reciprocal-mating and suck behaviour of *Macrostomum hamatum* described in the text and in Figure 2 and 3 (For a high-quality movie, please see https://zenodo.org/records/6976430). This detailed observation movie was made of a mating chamber that had seventeen worms distributed in six drops, but it is not clear which close-up movies correspond to which drops/worms. After the close-up movie, this mating chamber and worms were used for recording another longer movie Macrostomum hamatum 4.mov.

Additional file 2. Movie S2. Behavioural instances in reciprocally mating species where the mating posture deviates from that observed in *M. lignano *. Note that the movies are recorded at one frame per second, but played back at a rate of ten frames per second, i.e. a 1:10 time lapse. See the inset time stamp for the absolute timing (full movies will be available online in a public data repository). A. *Macrostomum* sp. 59 In this movie clip, there are two drops with three worms in each drop. In the left drop, two worms, soon after the start of the movie, start mating reciprocally. This is visible as the two worms being intertwined, such that the female antrum was accessible to the partner's stylet and vice-versa. This was followed by brief sucks by both individuals, and then they separated. After some time, they briefly mate reciprocally again, followed by both individuals sucking. B. *Macrostomum* sp. 61 In this movie clip, there are two drops with three worms in each drop. In the left drop, two worms mate reciprocally by intertwining. However, they then partially untwined, such that they resembled a pretzel with both worms’ anterior part facing in the same direction.

Additional file 3. Movie S3. Behavioural instances that could possibly represent cases of hypodermic insemination. Note that the movies are recorded at one frame per second, but played back at a rate of ten frames per second, i.e. a 1:10 time lapse. See the inset time stamp for the absolute timing (full movies will be available online in a public data repository). A. *Macrostomum* sp. 1 In this movie clip, there is a single drop with two worms. Shortly after the movie begins, one of the worms seems to attach to the tail plate of the other worm by its posterior region (where the stylet or male intromittent organ would be located). This could possibly be hypodermic insemination. After some time, this phenomenon is again rapidly repeated. B. *M. gabriellae* In this movie clip, there is a single drop with two worms that are rapidly moving. They come into contact briefly, when one of the worms reverses its direction rapidly, and puts its posterior region (where the stylet or male intromittent organ would be located) in touch with of the partner’s mid-region. This could potentially be hypodermic insemination.

Additional file 4. Table S1. Data on observation records of reproductive behaviour and the inferred mating syndrome in 64 species in the genus *Macrostomum *. Table S2. Reciprocal mating behaviour for species that differ from the canonical mating behaviour originally described for *Macrostomum lignano* . Table S3. For the duration and frequency of both reciprocal mating and suck behaviour, we determined the model fit of different character evolution models. Given are the values of σ 2 (Brownian rate parameter), α (selection strength parameter), a (rate of evolutionary change parameter), and λ (phylogenetic signal) for the different models, as well as the sample-size corrected Akaike Information Criterion (AICc) and the relevant Akaike weights (ω 1 ). Table S4. Marginal likelihoods and Bayes Factor values of the independent and dependent models (three independent runs each) examining the correlated evolution between a) the presence/absence of reciprocal mating and the suck behaviour, and b) the presence/absence of reciprocal mating and the reciprocal inferred mating syndrome, for the entire dataset and for the reduced dataset (i.e. excluding species with < 21 h observation time, see Methods). Summary of the PGLS and linear regression results for the association between aspects of reciprocal mating and the suck behaviour for the reduced dataset (20 species, i.e. excluding species in which mating or suck had only been observed in one replicate). Figure S1. (A) High-resolution PDF version of Figure 2(available separately) . (B) High-resolution PDF version of Figure 3 (available separately). Figure S2. The posterior distributions of the rate parameters (x-axis) for the different transitions in the dependent model of character state evolution. Figure S3. Plot showing the number of hours for which each species was observed, split by the four different combinations of behaviours that either were or were not observed (i.e. the reciprocal mating and suck behaviour are either absent or present), for species with the different inferred mating syndromes (hypodermic, intermediate, and reciprocal).

## Data Availability

All data in this manuscript will be deposited online in a public data repository. Data in this manuscript has been deposited online on Dryad (10.5061/dryad.n8pk0p35b) and Zenodo (https://zenodo.org/records/6976430, https://zenodo.org/records/6957067).

## Abbreviations

BF: Bayes Factor
PGLS: Phylogenetic generalised least-squares
RJ MCMC: Reversible Jump Markov Chain Monte Carlo
