# Supplementary material for: Evolution and co-evolution of the suck behaviour, a postcopulatory female resistance trait that manipulates received ejaculate
Source: BMC Biol. 2025 Mar 26;23:87. doi: 10.1186/s12915-025-02171-5 (PMC11948766; doi:10.1186/s12915-025-02171-5)

**Additional file 1**

**Table S1.** Data on observation records of reproductive behaviour and the inferred mating syndrome in 64 species in the genus *Macrostomum*. The number of worms is the total number of worms observed for that species across all replicate drops. Similarly, the number of hours is the total number of movie hours observed for that species across all replicate drops. The number of replicate drops with reciprocal mating and suck signify the number of drops in which we observed a reciprocal mating and suck, respectively, for that species. For each replicate drop, we obtained a standardized value for the frequency of matings and sucks by dividing by the number of worms and hours. For each species, we then averaged the value over all replicate drops. This gave us comparable standardised values across all species. In certain species, we observed no reciprocal mating and/or suck, and this is denoted by ‘-’. Note that an absence of observations of reciprocal mating and/or suck may be due to a lack of sufficient data for observing the behaviour. It does therefore not necessarily imply the presence of hypodermic insemination or the absence of the suck behaviour in these species. Moreover, *Macrostomum* sp. 65, *Macrostomum* sp. 67 and *Macrostomum* sp. 117 are now formally named as *Macrostomum* *pellitum,* *Macrostomum* *longispermatum*, and *Macrostomum gracilistylum,* respectively (Brand, 2023).

| Species | No. of worms | No. of hours | No. of replicate drops | No. of replicate drops with reciprocal mating | Reciprocal mating frequency (h^-1^ worm^-1^) (Mean ± sd) | Reciprocal mating duration (s)  (Mean ± sd) | No. of replicate drops with suck | Suck frequency (h^-1^ worm^-1^) (Mean ± sd) | Suck duration (s) (Mean ± sd) | Inferred mating syndrome |
| --- | --- | --- | --- | --- | --- | --- | --- | --- | --- | --- |
| *M. axi* | 180 | 491.34 | 67 | 23 | 0.16 ± 0.08 | 200.87 ± 58.77 | 26 | 0.22 ± 0.15 | 14.21 ± 2.83 | Reciprocal |
| *M. balticum* | 18 | 48.00 | 9 | 1 | 0.19 | 35.33 | 1 | 0.31 | 12.4 | Reciprocal |
| *M. baoanense* | 20 | 26.54 | 10 | 6 | 0.42 ± 0.11 | 30.28 ± 10.65 | 3 | 0.31 ± 0.06 | 8.33 ± 1.15 | Reciprocal |
| *M. clavituba* | 88 | 235.03 | 38 | 26 | 0.47 ± 0.44 | 5.41 ± 1.76 | 21 | 0.4 ± 0.30 | 5.43 ± 0.52 | Reciprocal |
| *M. cliftonense* | 36 | 42.00 | 18 | 12 | 1.23 ± 1.29 | 35.59 ± 14.82 | 11 | 1.20 ± 1.03 | 11.74 ± 1.85 | Reciprocal |
| *M. curvituba* | 114 | 62.00 | 35 | 15 | 0.58 ± 0.49 | 6.64 ± 2.24 | 13 | 0.58 ± 0.47 | 8.02 ± 1.53 | Reciprocal |
| *M. distinguendum* | 26 | 31.00 | 9 | 1 | 0.1 | 15 | - | - | - | Hypodermic |
| *M. finnlandense* | 50 | 108.00 | 18 | - | - | - | 1 | 0.05 | 6 | Hypodermic |
| *M. gabriellae* | 80 | 80.00 | 40 | - | - | - | - | - | - | Hypodermic |
| *M. gieysztori* | 13 | 20.00 | 4 | 1 | 0.3 | 5.25 | - | - | - | Reciprocal |
| *M. hamatum* | 63 | 192.00 | 28 | 9 | 0.24 ± 0.23 | 52.71 ± 19.22 | 11 | 0.61 ± 0.77 | 11.35 ± 1.58 | Reciprocal |
| *M. hystricinum* | 7 | 8.00 | 1 | - | - | - | - | - | - | Hypodermic |
| *M. hystricinum_d* | 24 | 24.00 | 12 | - | - | - | - | - | - | Hypodermic |
| *M. hystrix* | 145 | 210.98 | 64 | - | - | - | - | - | - | Hypodermic |
| *M. inductum* | 16 | 32.00 | 6 | - | - | - | 1 | 0.125 | 8 | Reciprocal |
| *M. janickei* | 120 | 58.73 | 60 | 56 | 3.74 ± 1.92 | 81.94 ± 30.1 | 39 | 1.63 ± 1.08 | 9.74 ± 1.71 | Reciprocal |
| *M. karlingi* | 5 | 16.00 | 1 | - | - | - | 1 | 0.01 | 8 | Reciprocal |
| *M. kepneri* | 6 | 12.00 | 2 | - | - | - | 2 | 0.14 ± 0.03 | 8 ± 0 | Reciprocal |
| *M. lignano* | 114 | 29.95 | 57 | 54 | 7.82 ± 4.37 | 14.99 ± 4.11 | 50 | 3.70 ± 2.54 | 8.21 ± 1.71 | Reciprocal |
| *M. longituba* | 42 | 65.43 | 16 | 9 | 1.92 ± 2.38 | 36.31 ± 11.08 | 8 | 0.57 ± 0.55 | 10.14 ± 3.51 | Reciprocal |
| *M. mediterraneum* | 26 | 20.88 | 11 | 9 | 3.13 ± 1.56 | 40.60 ± 11.14 | 5 | 0.9 ± 0.91 | 13.62 ± 1.11 | Reciprocal |
| *M. minutum* | 33 | 29.00 | 7 | - | - | - | - | - | - | Reciprocal |
| *M. mirumnovem* | 273 | 560.20 | 117 | 11 | 0.28 ± 0.11 | 940.5 ± 236.51 | 15 | 0.32 ± 0.23 | 15.71 ± 2.28 | Reciprocal |
| *M. mystrophorum* | 9 | 21.69 | 4 | - | - | - | - | - | - | Reciprocal |
| *M. paradoxum* | 138 | 194.00 | 40 | 1 | 0.1 | 15 | 13 | 0.08 ± 0.06 | 5.69 ± 1.33 | Reciprocal |
| *M. poznaniense* | 36 | 178.31 | 13 | 1 | 0.03 | 20 | - | - | - | Reciprocal |
| *M. pusillum* | 98 | 84.99 | 27 | - | - | - | - | - | - | Hypodermic |
| *M. quiritium* | 18 | 25.78 | 3 | - | - | - | - | - | - | Reciprocal |
| *M. retortum* | 10 | 37.12 | 4 | - | - | - | - | - | - | Reciprocal |
| *M. rostratum* | 28 | 26.75 | 8 | 1 | 0.02 | 18 | 3 | 0.14 ± 0.09 | 5.58 ± 0.37 | Hypodermic |
| *M. spirale* | 249 | 644.38 | 109 | 11 | 0.27 ± 0.25 | 294.12 ± 203.84 | 10 | 0.65 ± 0.84 | 16.08 ± 4.32 | Reciprocal |
| *M. tenuicauda* | 32 | 59.26 | 15 | 9 | 0.20 ± 0.16 | 42.5 ± 17.67 | 6 | 0.13 ± 0.07 | 8.8 ± 1.52 | Reciprocal |
| *M. tuba* | 43 | 146.41 | 18 | 12 | 0.57 ± 0.68 | 112.42 ± 46.32 | 5 | 0.21 ± 0.31 | 6.78 ± 1.59 | Reciprocal |
| *Macrostomum* sp. 1 | 12 | 10.01 | 6 | - | - | - | - | - | - | Hypodermic |
| *Macrostomum* sp. 2 | 8 | 12.00 | 2 | 2 | 0.08 ± 0.07 | 8 ± 1.41 | 2 | 0.37 ± 0.35 | 10.3 ± 0.99 | Reciprocal |
| *Macrostomum* sp. 4 | 16 | 48.89 | 4 | - | - | - | - | - | - | Reciprocal |
| *Macrostomum* sp. 16 | 2 | 1.33 | 1 | - | - | - | - | - | - | Reciprocal |
| *Macrostomum* sp. 17 | 6 | 6.00 | 2 | - | - | - | 1 | 0.67 | 4.75 | Reciprocal |
| *Macrostomum* sp. 22 | 12 | 12.00 | 6 | - | - | - | - | - | - | Hypodermic |
| *Macrostomum* sp. 30 | 18 | 27.92 | 6 | 1 | 0.04 | 1002 | 3 | 0.11 ± 0.08 | 10.67 ± 2.52 | Reciprocal |
| *Macrostomum* sp. 34 | 3 | 3.00 | 1 | - | - | - | - | - | - | Hypodermic |
| *Macrostomum* sp. 36 | 8 | 8.00 | 3 | - | - | - | - | - | - | Reciprocal |
| *Macrostomum* sp. 42 | 91 | 172.74 | 40 | 6 | 0.45 ± 0.23 | 802.55 ± 1212.71 | 3 | 0.28 ± 0.21 | 12 ± 1 | Reciprocal |
| *Macrostomum* sp. 43 | 2 | 6.02 | 1 | 1 | 0.08 | 4609 | - | - | - | Reciprocal |
| *Macrostomum* sp. 47 | 10 | 13.09 | 5 | - | - | - | - | - | - | Hypodermic |
| *Macrostomum* sp. 48 | 10 | 9.67 | 5 | - | - | - | - | - | - | Hypodermic |
| *Macrostomum* sp. 49 | 10 | 10.91 | 5 | - | - | - | - | - | - | Hypodermic |
| *Macrostomum* sp. 55 | 14 | 26.20 | 5 | - | - | - | - | - | - | Reciprocal |
| *Macrostomum* sp. 59 | 12 | 12.00 | 4 | 4 | 1.11 ± 0.70 | 26.14 ± 6.02 | 4 | 0.5 ± 0.23 | 8.51 ± 0.64 | Reciprocal |
| *Macrostomum* sp. 61 | 12 | 12.00 | 4 | 3 | 0.15 ± 0.06 | 15.67 ± 4.93 | 1 | 0.22 | 8 | Reciprocal |
| *Macrostomum* sp. 62 | 18 | 15.35 | 6 | - | - | - | - | - | - | Hypodermic |
| *Macrostomum* sp. 64 | 18 | 18.00 | 6 | - | - | - | - | - | - | Hypodermic |
| *Macrostomum* *pellitum* | 12 | 12.00 | 4 | - | - | - | - | - | - | Reciprocal |
| *Macrostomum* sp. 66 | 12 | 9.33 | 4 | - | - | - | - | - | - | Reciprocal |
| *Macrostomum* *longispermatum* | 3 | 2.58 | 1 | 1 | 0.26 | 9 | - | - | - | Reciprocal |
| *Macrostomum* sp. 78 | 55 | 38.33 | 13 | - | - | - | - | - | - | Hypodermic |
| *Macrostomum* sp. 80 | 45 | 57.00 | 13 | - | - | - | - | - | - | Hypodermic |
| *Macrostomum* sp. 82 | 10 | 12.00 | 4 | - | - | - | - | - | - | Reciprocal |
| *Macrostomum* sp. 83 | 12 | 12.00 | 4 | - | - | - | - | - | - | Reciprocal |
| *Macrostomum* sp. 100 | 28 | 36.00 | 9 | - | - | - | - | - | - | Hypodermic |
| *Macrostomum* sp. 101 | 47 | 75.09 | 20 | - | - | - | 3 | 0.12 ± 0.05 | 9.67 ± 1.53 | Intermediate |
| *Macrostomum* sp. 103 | 55 | 116.93 | 27 | 3 | 0.29 ± 0.19 | 13.61 ± 5.25 | 7 | 0.13 ± 0.06 | 9.33 ± 0.99 | Reciprocal |
| *Macrostomum* sp. 108 | 30 | 46.36 | 15 | 9 | 0.67 ± 0.50 | 10.69 ± 3.51 | 8 | 1.32 ± 1.06 | 12.331 ± 3.02 | Reciprocal |
| *Macrostomum* *gracilistylum* | 45 | 243.77 | 17 | 3 | 0.27 ± 0.11 | 9.69 ± 1.03 | 5 | 0.66 ± 1.08 | 9.70 ± 0.88 | Reciprocal |

**Table S2.** Reciprocal mating behaviour for species that differ from the canonical mating behaviour originally described for *Macrostomum lignano*.

| Species | Reciprocal mating behaviour |
| --- | --- |
| *M. distinguendum* | The anterior part of both worms faced the same direction, while the posterior body was twisted at an angle such that the tail plates of the worms were in ventral contact with the female antrum accessible to the partner’s stylet and vice-versa, permitting the possibility of reciprocal transfer of ejaculate. |
| *M. paradoxum* | The entire body of the worms were intertwined with two turns of the body, with the tail plates in ventral contact, such that the female antrum was accessible to the partner's stylet and vice-versa, permitting the possibility of reciprocal transfer of ejaculate. After some time, they untwined slightly, and at this point they remained attached at their tail plates ventrally, but the rest of the body was unattached, facing opposite directions. |
| *M. tuba* | The posterior bodies of the worms were twisted at an angle such that the tail plates were in ventral contact with the female antrum accessible to the partner’s stylet and vice-versa, allowing reciprocal transfer of ejaculate*,* although the anterior ventral side of each worm remained attached to the substrate with the worm's heads pointing in opposite directions. |
| *Macrostomum* sp. 59 | Similar to *M. paradoxum* (see also Supplementary Movie S2A). |
| *Macrostomum* sp. 61 | The worms intertwined similar to M. *paradoxum*, but towards the end they only partially untwined, such that they resembled a pretzel with both worms’ anterior part facing in the same direction (see also Supplementary Movie S2B). |
| *Macrostomum* *longispermatum* | The worms were intertwined, with the tail plates in ventral contact such that the female antrum was accessible to the partner's stylet and vice-versa, permitting the possibility of reciprocal transfer of ejaculate. |

**Table S3.** For the duration and frequency of both reciprocal mating and suck behaviour (all log-transformed), we determined the model fit of different character evolution models (i.e. the Brownian motion, Ornstein–Uhlenbeck, Early-burst, and Lambda models). Given are the values of σ^2^ (Brownian rate parameter), α (selection strength parameter), *a* (rate of evolutionary change parameter), and λ (phylogenetic signal) for the different models, as well as the sample-size corrected Akaike Information Criterion (AICc) and the relevant Akaike weights (ω_ι_).

| Effect size | Brownian motion | | | Ornstein-Uhlenbeck | | | | Early-burst | | | | Lambda | | | |
| --- | --- | --- | --- | --- | --- | --- | --- | --- | --- | --- | --- | --- | --- | --- | --- |
|  | σ^2^ | AICc | ω_ι_ | σ^2^ | α | AICc | ω_ι_ | σ^2^ | a | AICc | ω_ι_ | λ | σ^2^ | AICc | ω_ι_ |
| Reciprocal mating frequency | 16.62 | 123.38 | 0.00 | 20.84 | 2.72 | 116.59 | 0.01 | 16.63 | -0.000001 | 125.85 | 0.00 | 0.50 | 4.38 | 106.34 | **0.99** |
| Reciprocal mating duration | 22.95 | 133.05 | 0.00 | 28.87 | 2.71 | 126.38 | 0.05 | 22.95 | -0.000001 | 135.52 | 0.00 | 0.46 | 6.85 | 120.38 | **0.95** |
| Suck frequency | 6.90 | 101.87 | 0.05 | 9.90 | 2.71 | 98.35 | 0.27 | 6.90 | -0.000001 | 104.33 | 0.01 | 0.67 | 3.22 | 96.58 | **0.66** |
| Suck duration | 0.45 | 17.62 | 0.10 | 0.68 | 2.72 | 15.49 | 0.28 | 0.45 | -0.000001 | 20.09 | 0.03 | 0.76 | 0.24 | 13.99 | **0.59** |

**Table S4.** Marginal likelihoods and Bayes Factor values of the independent and dependent models (three independent runs each) examining the correlated evolution between a) the presence/absence of reciprocal mating and the suck behaviour, and b) the presence/absence of reciprocal mating and the reciprocal inferred mating syndrome, for the entire dataset and for the reduced dataset (i.e. excluding species with < 21 h observation time, see Methods).

|  | | Entire dataset | | | Reduced dataset | | |
| --- | --- | --- | --- | --- | --- | --- | --- |
|  |  | Independent | Dependent | Bayes Factor | Independent | Dependent | Bayes Factor |
| a) Presence/absence of reciprocal mating and suck behaviour | Run 1 | -88.72 | -82.61 | 12.22 | -74.53 | -66.27 | 16.52 |
|  | Run 2 | -88.71 | -82.59 | 12.24 | -74.53 | -66.31 | 16.44 |
|  | Run 3 | -88.72 | -82.60 | 12.22 | -74.52 | -66.32 | 16.42 |
|  | **Average** | **-88.71** | **-82.60** | **12.23** | **-74.53** | **-66.30** | **16.46** |
| b) Presence/absence of reciprocal mating and reciprocal inferred mating syndrome | Run 1 | -67.85 | -64.54 | 6.62 | -63.63 | -60.47 | 6.31 |
|  | Run 2 | -67.86 | -64.51 | 6.67 | -63.64 | -60.52 | 6.24 |
|  | Run 3 | -67.85 | -64.56 | 6.57 | -63.63 | -60.52 | 6.20 |
|  | **Average** | **-67.85** | **-64.54** | **6.62** | **-63.63** | **-60.50** | **6.25** |

**Table S5.** Summary of the PGLS and linear regression results for the association between aspects of reciprocal mating and the suck behaviour for the reduced dataset (20 species, i.e. excluding species in which mating or suck had only been observed in one replicate). PGLS was done on log-transformed variables, and λ is the phylogenetic scaling parameter for the PGLS. Note that this reduced dataset is different from the reduced dataset of BayesTraits analysis above.

| Dependent variable | Predictor variable | λ | β±s.e. | t, df | P |
| --- | --- | --- | --- | --- | --- |
| Suck frequency | Reciprocal mating frequency | 0 | 0.52±0.12 | 4.45, 18 | **0.0003** |
| Suck duration | Reciprocal mating duration | 0.64 | 0.1±0.03 | 3.57, 18 | **0.002** |
| Reciprocal mating frequency | Reciprocal mating duration | 0.19 | -0.03±0.17 | -0.19, 19 | 0.84 |
| Suck frequency | Suck duration | 0.009 | 0.98±0.64 | 1.53, 23 | 0.14 |

**Figure S1**

1. High-resolution PDF version of Figure 3 (available separately)*.*
2. High-resolution PDF version of Figure 4 (available separately).

**Figure S2.** The posterior distributions of the rate parameters (x-axis) for the different transitions in the dependent model of character state evolution. Three separate runs were performed, represented by green, blue and purple. Note that since they are largely overlapping the different runs are often difficult to see in the figure. Correlated evolution between the presence/absence of reciprocal mating and the suck behaviour (A), and the reciprocal inferred mating syndrome (B). The panels show the transition rates and the Z value (in brackets, expressed as %) for the different transitions, where the Z value can be understood as the percentage of times the transition rate was set to zero. The different arrows represent different probabilities of transitions between the states: high probability (strong black arrows, Z value < 15%), moderate probability (thin black arrows, Z value 20-55%), and low probability (dashed black arrows, Z value > 85%).


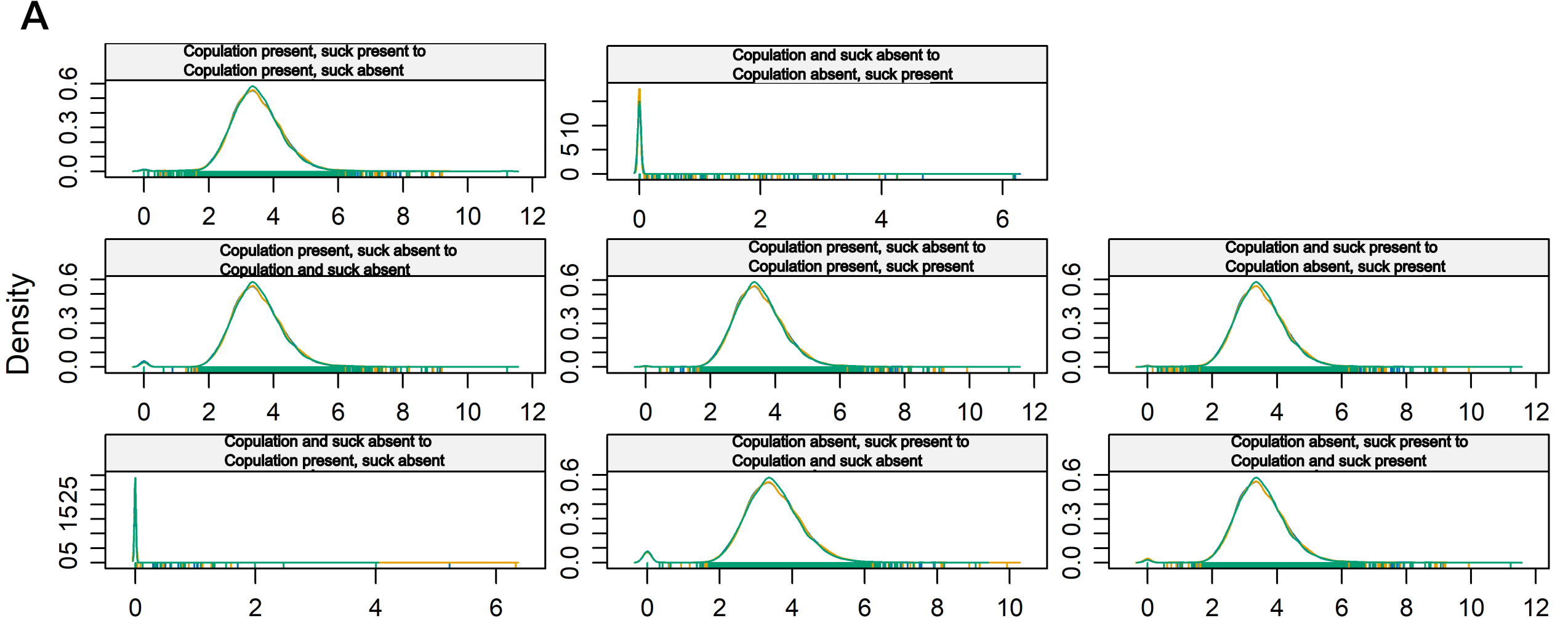


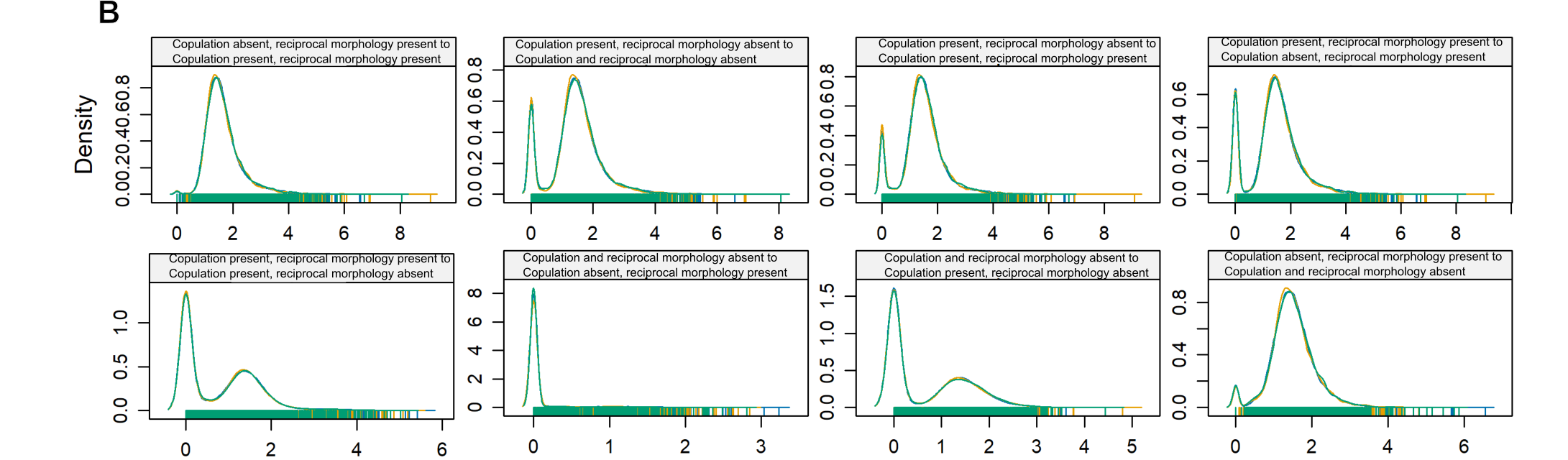


**Figure S3.** Plot showing the number of hours for which each species was observed, split by the four different combinations of behaviours that either were or were not observed (i.e. the reciprocal mating and suck behaviour are either absent or present), for species with the different inferred mating syndromes (hypodermic, intermediate, and reciprocal). Note that the few species that exhibited the hypodermic inferred mating syndrome, but showed either reciprocal mating or suck behaviour (or both) have been labelled.


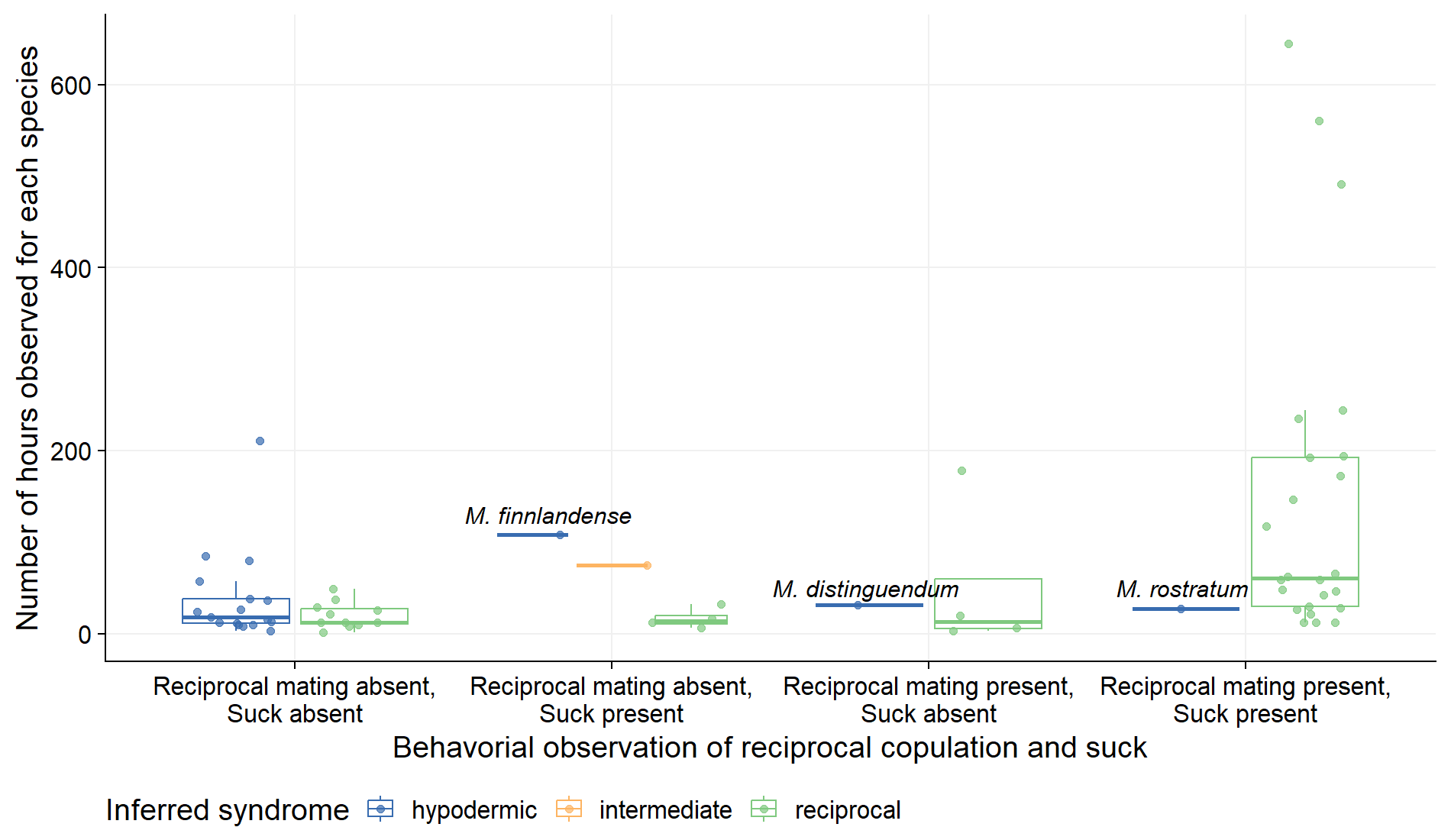

Supplement: Supplementary file 4 — Additional file 4. Table S1. Data on observation records of reproductive behaviour and the inferred mating syndrome in 64 species in the genus Macrostomum . Table S2. Reciprocal mating behaviour for species that differ from the canonical mating behaviour originally described for Macrostomum lignano . Table S3. For the duration and frequency of both reciprocal mating and suck behaviour, we determined the model fit of different character evolution models. Given are the values of σ 2 (Brownian rate parameter), α (selection strength parameter), a (rate of evolutionary change parameter), and λ (phylogenetic signal) for the different models, as well as the sample-size corrected Akaike Information Criterion (AICc) and the relevant Akaike weights (ω 1 ). Table S4. Marginal likelihoods and Bayes Factor values of the independent and dependent models (three independent runs each) examining the correlated evolution between a) the presence/absence of reciprocal mating and the suck behaviour, and b) the presence/absence of reciprocal mating and the reciprocal inferred mating syndrome, for the entire dataset and for the reduced dataset (i.e. excluding species with < 21 h observation time, see Methods). Summary of the PGLS and linear regression results for the association between aspects of reciprocal mating and the suck behaviour for the reduced dataset (20 species, i.e. excluding species in which mating or suck had only been observed in one replicate). Figure S1. (A) High-resolution PDF version of Figure 2(available separately) . (B) High-resolution PDF version of Figure 3 (available separately). Figure S2. The posterior distributions of the rate parameters (x-axis) for the different transitions in the dependent model of character state evolution. Figure S3. Plot showing the number of hours for which each species was observed, split by the four different combinations of behaviours that either were or were not observed (i.e. the reciprocal mating and suck behaviour are either abse [file 12915_2025_2171_MOESM4_ESM.zip › Additional file 4.docx]
