# Supplementary figures and images for: Evolution and co-evolution of the suck behaviour, a postcopulatory female resistance trait that manipulates received ejaculate
Source: BMC Biol. 2025 Mar 26;23:87. doi: 10.1186/s12915-025-02171-5 (PMC11948766; doi:10.1186/s12915-025-02171-5)

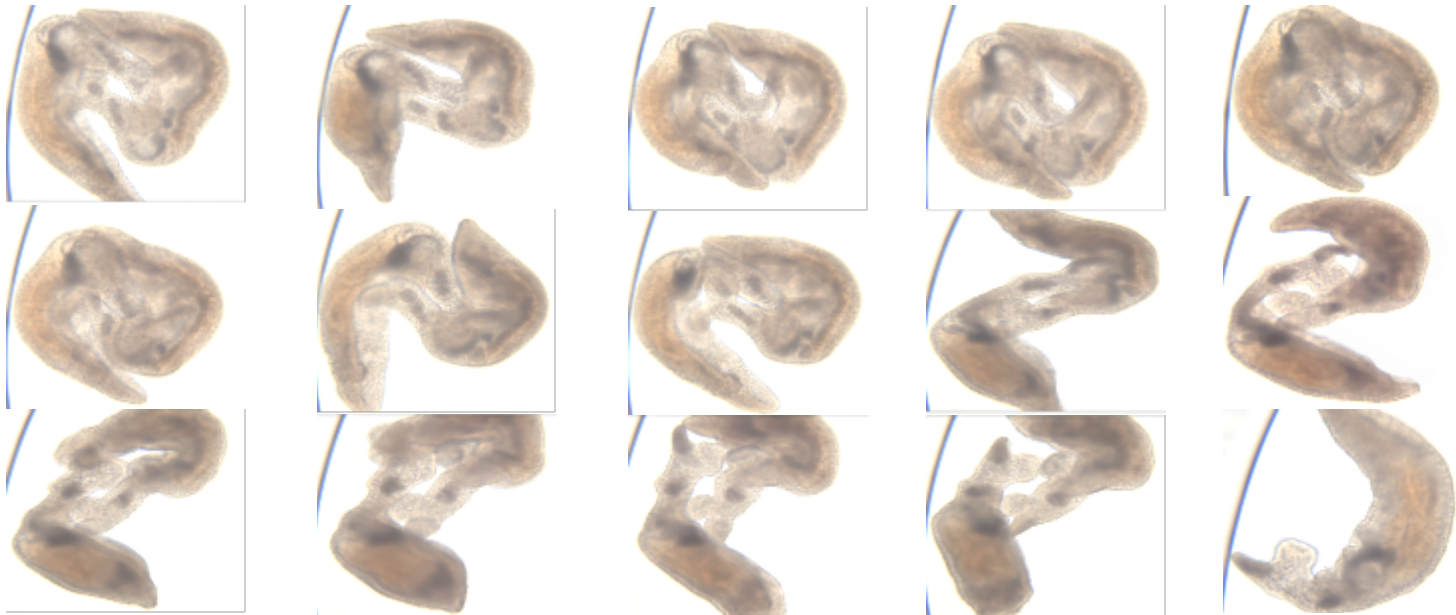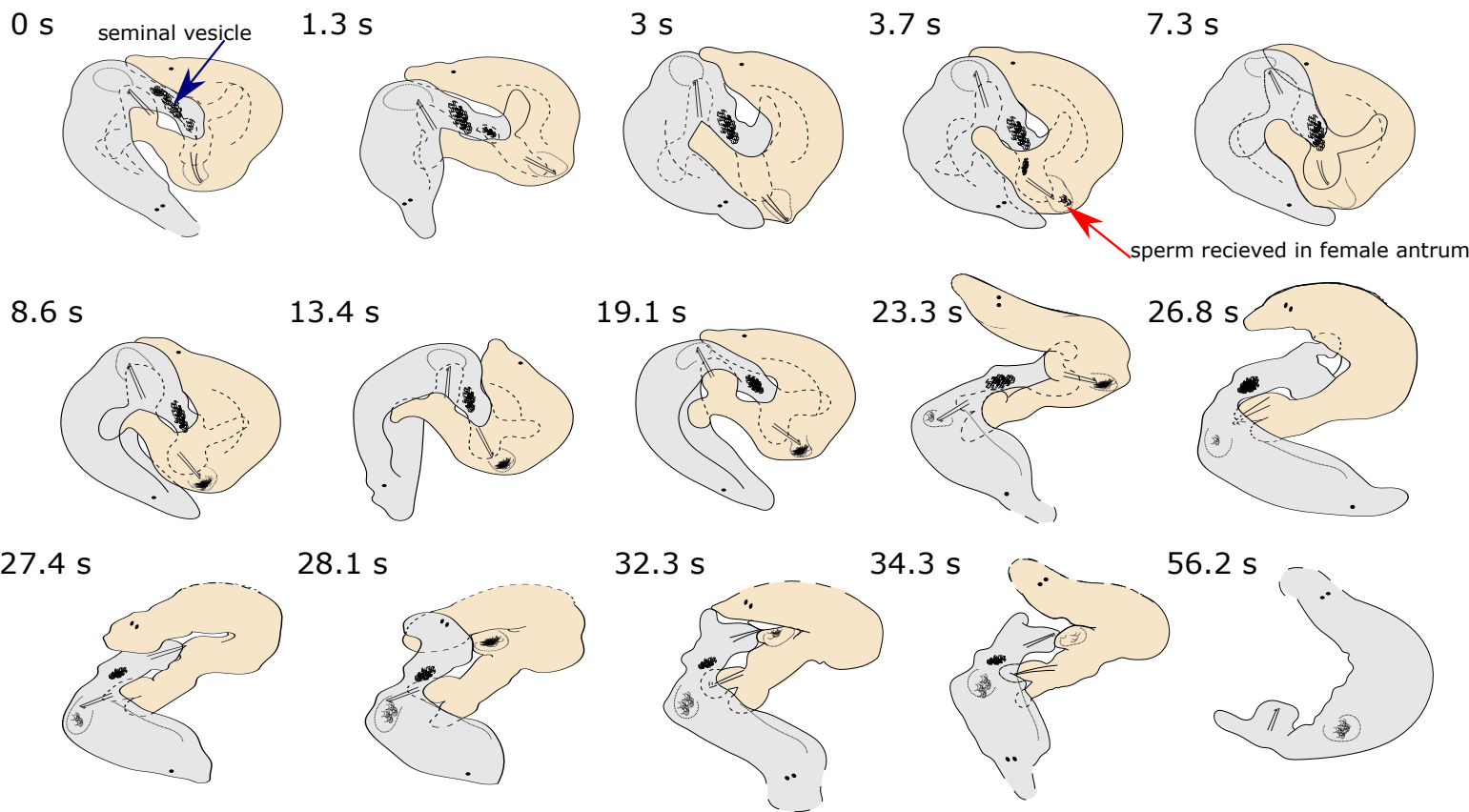

Supplement: Supplementary file 4 — Additional file 4. Table S1. Data on observation records of reproductive behaviour and the inferred mating syndrome in 64 species in the genus Macrostomum . Table S2. Reciprocal mating behaviour for species that differ from the canonical mating behaviour originally described for Macrostomum lignano . Table S3. For the duration and frequency of both reciprocal mating and suck behaviour, we determined the model fit of different character evolution models. Given are the values of σ 2 (Brownian rate parameter), α (selection strength parameter), a (rate of evolutionary change parameter), and λ (phylogenetic signal) for the different models, as well as the sample-size corrected Akaike Information Criterion (AICc) and the relevant Akaike weights (ω 1 ). Table S4. Marginal likelihoods and Bayes Factor values of the independent and dependent models (three independent runs each) examining the correlated evolution between a) the presence/absence of reciprocal mating and the suck behaviour, and b) the presence/absence of reciprocal mating and the reciprocal inferred mating syndrome, for the entire dataset and for the reduced dataset (i.e. excluding species with < 21 h observation time, see Methods). Summary of the PGLS and linear regression results for the association between aspects of reciprocal mating and the suck behaviour for the reduced dataset (20 species, i.e. excluding species in which mating or suck had only been observed in one replicate). Figure S1. (A) High-resolution PDF version of Figure 2(available separately) . (B) High-resolution PDF version of Figure 3 (available separately). Figure S2. The posterior distributions of the rate parameters (x-axis) for the different transitions in the dependent model of character state evolution. Figure S3. Plot showing the number of hours for which each species was observed, split by the four different combinations of behaviours that either were or were not observed (i.e. the reciprocal mating and suck behaviour are either abse [file 12915_2025_2171_MOESM4_ESM.zip › Additional file 4. Figure S1A.pdf]

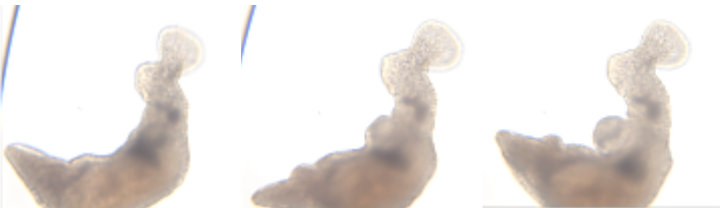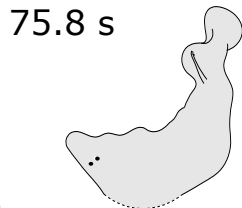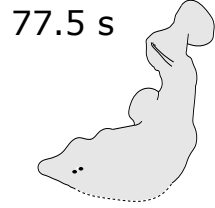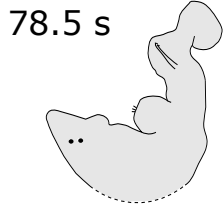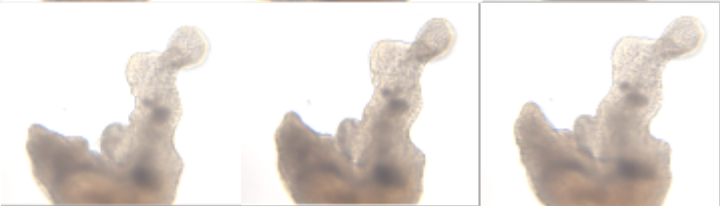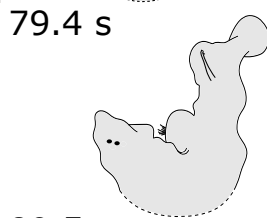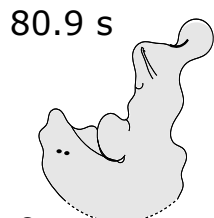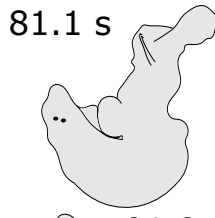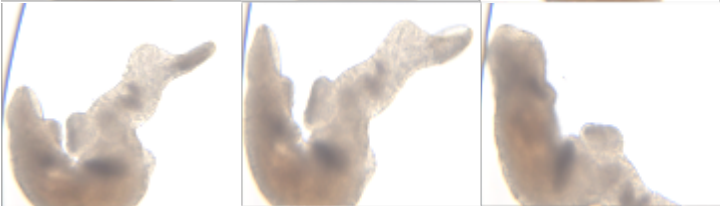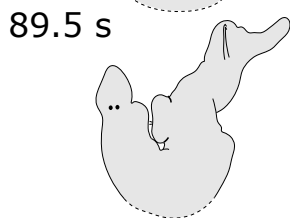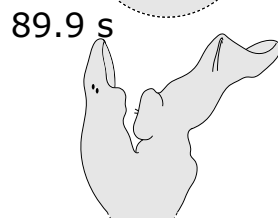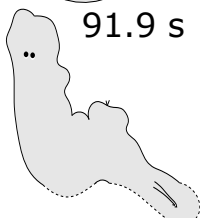

Supplement: Supplementary file 4 — Additional file 4. Table S1. Data on observation records of reproductive behaviour and the inferred mating syndrome in 64 species in the genus Macrostomum . Table S2. Reciprocal mating behaviour for species that differ from the canonical mating behaviour originally described for Macrostomum lignano . Table S3. For the duration and frequency of both reciprocal mating and suck behaviour, we determined the model fit of different character evolution models. Given are the values of σ 2 (Brownian rate parameter), α (selection strength parameter), a (rate of evolutionary change parameter), and λ (phylogenetic signal) for the different models, as well as the sample-size corrected Akaike Information Criterion (AICc) and the relevant Akaike weights (ω 1 ). Table S4. Marginal likelihoods and Bayes Factor values of the independent and dependent models (three independent runs each) examining the correlated evolution between a) the presence/absence of reciprocal mating and the suck behaviour, and b) the presence/absence of reciprocal mating and the reciprocal inferred mating syndrome, for the entire dataset and for the reduced dataset (i.e. excluding species with < 21 h observation time, see Methods). Summary of the PGLS and linear regression results for the association between aspects of reciprocal mating and the suck behaviour for the reduced dataset (20 species, i.e. excluding species in which mating or suck had only been observed in one replicate). Figure S1. (A) High-resolution PDF version of Figure 2(available separately) . (B) High-resolution PDF version of Figure 3 (available separately). Figure S2. The posterior distributions of the rate parameters (x-axis) for the different transitions in the dependent model of character state evolution. Figure S3. Plot showing the number of hours for which each species was observed, split by the four different combinations of behaviours that either were or were not observed (i.e. the reciprocal mating and suck behaviour are either abse [file 12915_2025_2171_MOESM4_ESM.zip › Additional file 4. Figure S1B.pdf]
